# Ejaculatory Abstinence Affects the Sperm Quality in Normozoospermic Men—How Does the Seminal Bacteriome Respond?

**DOI:** 10.3390/ijms24043503

**Published:** 2023-02-09

**Authors:** Eva Tvrdá, Michal Ďuračka, Filip Benko, Anton Kováčik, Daniel Lovíšek, Eliška Gálová, Jana Žiarovská, Marianna Schwarzová, Miroslava Kačániová

**Affiliations:** 1 Institute of Biotechnology, Faculty of Biotechnology and Food Sciences, Slovak University of Agriculture in Nitra, Tr. A. Hlinku 2, 94976 Nitra, Slovakia; 2 AgroBioTech Research Centre, Slovak University of Agriculture in Nitra, Tr. A. Hlinku 2, 94976 Nitra, Slovakia; 3 Institute of Applied Biology, Faculty of Biotechnology and Food Sciences, Slovak University of Agriculture in Nitra, Tr. A. Hlinku 2, 94976 Nitra, Slovakia; 4 Department of Genetics, Faculty of Natural Sciences, Comenius University, Ilkovičova 6, Mlynská Dolina, 84215 Bratislava, Slovakia; 5 Institute of Plant and Environmental Sciences, Faculty of Agrobiology and Food Resources, Slovak University of Agriculture, Tr. A. Hlinku 2, 94976 Nitra, Slovakia; 6 Department of Fruit Science, Viticulture and Enology, Faculty of Horticulture and Landscape Engineering, Slovak University of Agriculture, Tr. A. Hlinku 2, 94976 Nitra, Slovakia; 7 Department of Bioenergetics, Food Analysis and Microbiology, Institute of Food Technology and Nutrition, University of Rzeszow, Cwiklinskiej 1, 35-601 Rzeszow, Poland

**Keywords:** ejaculatory abstinence, semen quality, bacteria, inflammation, oxidative stress, apoptosis

## Abstract

This study was designed to describe bacterial profiles of ejaculates collected following a long and short ejaculatory abstinence set in the context of changes in the conventional, oxidative, and immunological characteristics of semen. Two specimens were collected in succession from normozoospermic men (*n* = 51) following 2 days and 2 h, respectively. Semen samples were processed and analyzed according to the World Health Organization (WHO) 2021 guidelines. Afterwards, sperm DNA fragmentation, mitochondrial function, levels of reactive oxygen species (ROS), total antioxidant capacity, and oxidative damage to sperm lipids and proteins were evaluated in each specimen. Selected cytokine levels were quantified using the ELISA method. Bacterial identification by matrix-assisted laser desorption/ionization time-of-flight (MALDI-TOF) mass spectrometry revealed that samples collected following two days of abstinence presented with a higher bacterial load and diversity, and a greater prevalence of potentially uropathogenic bacteria including *Escherichia coli*, *Staphylococcus aureus* and *Enterococcus faecalis*. Only staphylococci and *Escherichia coli* remained present in specimens obtained after 2 h of abstinence. Whilst all samples accomplished the criteria set by WHO, a significantly higher motility (*p* < 0.05), membrane integrity (*p* < 0.05), mitochondrial membrane potential (*p* < 0.05), and DNA integrity (*p* < 0.0001) were detected following 2 h of ejaculatory abstinence. On the other hand, significantly higher ROS levels (*p* < 0.001), protein oxidation (*p* < 0.001), and lipid peroxidation (*p* < 0.01) accompanied by significantly higher concentrations of tumor necrosis factor alpha (*p* < 0.05), interleukin-6 (*p* < 0.01), and interferon gamma (*p* < 0.05) were observed in specimens collected after two days of abstinence. It may be summarized that shorter ejaculatory abstinence does not compromise sperm quality in normozoospermic men, while it contributes to a decreased occurrence of bacteria in semen which is accompanied by a lower probability of damage to spermatozoa by ROS or pro-inflammatory cytokines.

## 1. Introduction

The decline of semen quality even in normozoospermic men has become a major global public health issue, causing emotional, financial, and social burden on the male [1]. Numerous efforts have been made to elucidate deeper causes of male reproductive dysfunction and to develop new strategies to improve male subfertility. However, few effective management options are currently available to prevent or counteract the deterioration of male reproductive potential, since sperm production and maturation may be affected by a multitude of genetic, environmental, metabolic, or physiological factors [2]. No matter the etiology underlying a decreased sperm vitality and fertilization ability, semen analysis as a golden standard of andrology serves to provide basic information on the function of male gonads and accessory glands [3]. Over the years many andrologists have been continually assessing and remodeling the cut-off values for different semen quality parameters in reference to male fertility. All of these culminate in general recommendations summarized in the manual for the examination and processing of human semen issued by the World Health Organization (WHO), which defines optimal sperm parameters with reference to pregnancy outcomes [4,5].

Among the factors that may affect routine semen analysis is the duration of ejaculatory abstinence. According to WHO, two to seven days of abstinence is recommended when evaluating the quality of semen specimens [5]. Longer abstinence intervals generally lead to a poorer semen quality, since spermatozoa tend to accumulate in the efferent ducts, then overflow into the urethra and are flushed out in urine [6]. Whilst longer ejaculatory abstinence will increase the semen volume and sperm count, the resulting sperm motility and viability may be lower [7,8,9]. Furthermore, sperm accumulation in the epididymis may increase the risks for oxidative damage to male gametes due to increased reactive oxygen species (ROS) production necessary for sperm maturation [10,11]. Finally, spermatozoa collected following longer abstinence periods may present with increased alterations to the sperm DNA [10,12,13] and chromatin quality [14], considered to be crucial for the success of assisted reproductive technologies (ARTs) that currently rely on the sperm quality rather than quantity [15]. As such, the selection of the most appropriate ejaculatory abstinence period may be helpful in optimizing the sperm quantity and quality for prospectively planned ARTs [11,13]. In the meantime, scientific outcomes gathered from recent studies suggest that abstinence intervals as short as one day may lead to a better sperm motility and viability without dropping below the WHO cut-off values for sperm concentration [8,9,11,13,16]. Furthermore, semen collected following short abstinence periods may present with lower ROS levels and sperm DNA damage [11,12,13,16]. Several reports indicate that shortening the abstinence time to even a few hours could be beneficial for the overall sperm quality, particularly if the semen sample will be processed further for ARTs [11,17,18]. 

Despite a convincing body of evidence suggesting the potential of short ejaculatory abstinence in improving the sperm quality, its impact on the changes in the bacterial profiles of semen have been by and large overlooked. In the past, samples collected from the reproductive tract of clinically healthy males were considered free from bacteria, which has resulted in the male reproductive microbiome not being well described [19]. Yet, recent studies have found that bacteriocenoses are a normal part of the male urogenital system and body fluids, including semen and urine [20,21,22,23]. Urotypes dominated by *Enterococcus*, *Shigella*, or *Citrobacter* have been previously found in the urinary tract of healthy men [24]. At the same time, *Streptococcus* and *Corynebacterium* have been described as predominant representatives of the urobiome by several studies [25,26]. *Lactobacillus* and *Pseudomonas* genera have also been identified as members of the male microbiota [27]. Other frequently occurring genera in the male urinary microbiome include coagulase-negative staphylococci and *Eubacterium* [28]. Moreover, bacteria found in semen may also stem from the male genital mucosa which has been previously reported to host *Finegoldia*, *Staphylococcus*, *Prevotella*, *Corynebacterium*, *Anaerococcus*, or *Peptoniphilus* [29].

As matrix-assisted laser desorption/ionization-time of flight (MALDI-TOF) mass spectrometry (MS) and 16S ribosomal RNA sequencing have been applied to study the human microbiome, associations between the bacterial profiles of semen and sperm quality have been gradually revealed [21,22,30,31]. Bacteria may damage male reproductive cells either by direct cell-to-cell interactions through adhesion [32] leading to sperm immobilization and agglutination [33,34,35] or by the release of cytotoxic endotoxins [36]. Oxidative stress and leukocytospermia as side-effects of bacteriospermia may equally contribute to a lower semen quality [22,37,38]. Finally, local immune reaction triggered by bacterial presence may be accompanied by the release of pro-inflammatory cytokines that may exhibit detrimental effects on the sperm structure and function [22,38,39]. As such, rapid microbial screening methods should become routine in practical andrology to quickly unravel eventual damage to male gametes during semen processing and storage or to prevent the transmission of potential uropathogens to the female if such a compromised sample is used for ARTs. 

In view of the current lack of information of the ejaculatory abstinence-associated changes in the seminal bacteriome and its implications for semen quality, this study was designed to assess the bacterial profiles of ejaculates collected from normozoospermic men following a long (two days) and short (two h) ejaculatory abstinence. These variations were furthermore set in the context of changes observed in the conventional and functional sperm parameters, oxidative, and immunological characteristics of semen. 

## 2. Results

### 2.1. Semen Quality

**Figure 1:**
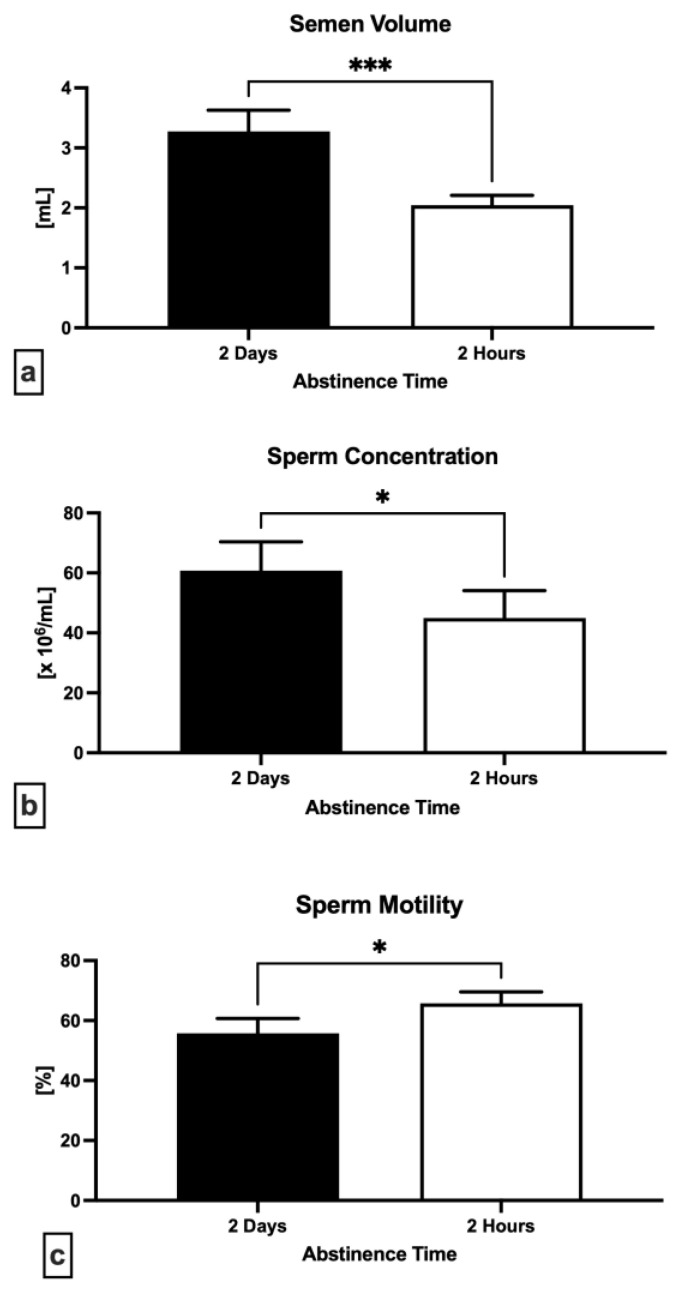
Variations in the semen volume (**a**), sperm concentration (**b**) and motility (**c**) following 2 days and 2 h of ejaculatory abstinence. * *p* < 0.05; *** *p* < 0.001.

Figure 1a reveals a significant decrease of semen volume following 2 h in comparison to 2 days (*p* = 0.0003). A significant decrease in the sperm concentration following short abstinence time was observed as well (*p* = 0.0127; Figure 1b). Inversely, a significantly increased sperm motility was recorded following 2 h of abstinence when compared to 2 days (*p* = 0.0288; Figure 1c). 

**Figure 2:**
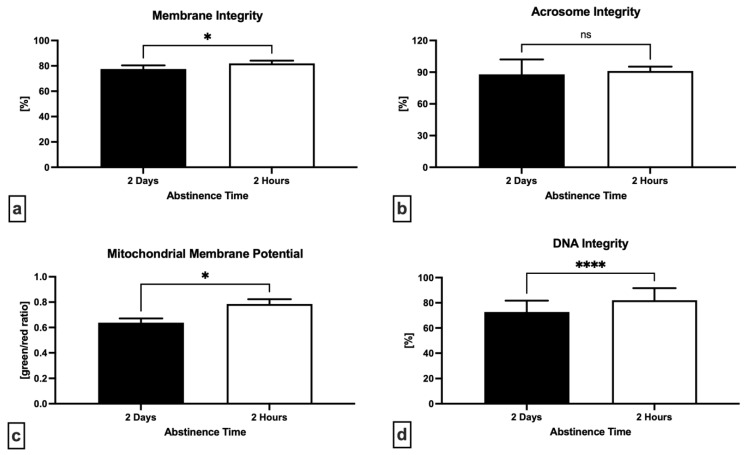
Variations in the membrane integrity (**a**), acrosome integrity (**b**), mitochondrial membrane potential (**c**) and DNA integrity (**d**) following 2 days and 2 h of ejaculatory abstinence. * *p* < 0.05; **** *p* < 0.0001; ns—non-significant.

A significantly higher sperm membrane integrity (*p* = 0.0301; Figure 2a) and mitochondrial membrane potential (*p* = 0.0150; Figure 2c) were found after short abstinence period when compared with long abstinence. The proportion of spermatozoa with an intact DNA molecule was significantly higher following a short abstinence (*p* < 0.0001; Figure 2d), whereas no significant differences were found in the case of acrosome integrity (*p* = 0.5824; Figure 2b). 

### 2.2. Oxidative Profile

**Figure 3:**
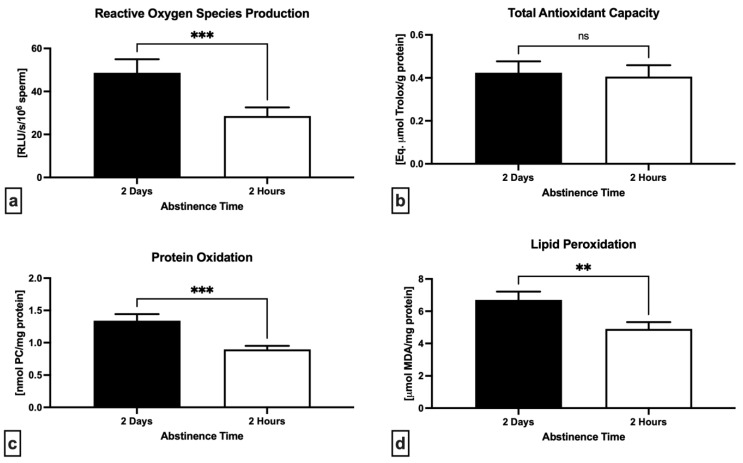
Variations in the levels of reactive oxygen species (ROS) in neat semen (**a**), total antioxidant capacity of the seminal plasma (**b**), oxidative damage to the proteins (**c**) and lipids (**d**) assessed in the sperm lysates following 2 days and 2 h of ejaculatory abstinence. ** *p* < 0.01; *** *p* < 0.001; ns—non-significant.

A significant decrease was observed in the levels of reactive oxygen species (*p* = 0.0001; Figure 3a) as well as protein carbonyls (*p* = 0.0003; Figure 3c) and malondialdehyde (*p* = 0.0090; Figure 3d) in semen specimens collected following 2 h in comparison to 2 days. Furthermore, a slight although non-significant decline of the total antioxidant capacity was noted following short abstinence in comparison with long abstinence (*p* = 0.7079; Figure 3b).

### 2.3. Immunological Profile

**Figure 4:**
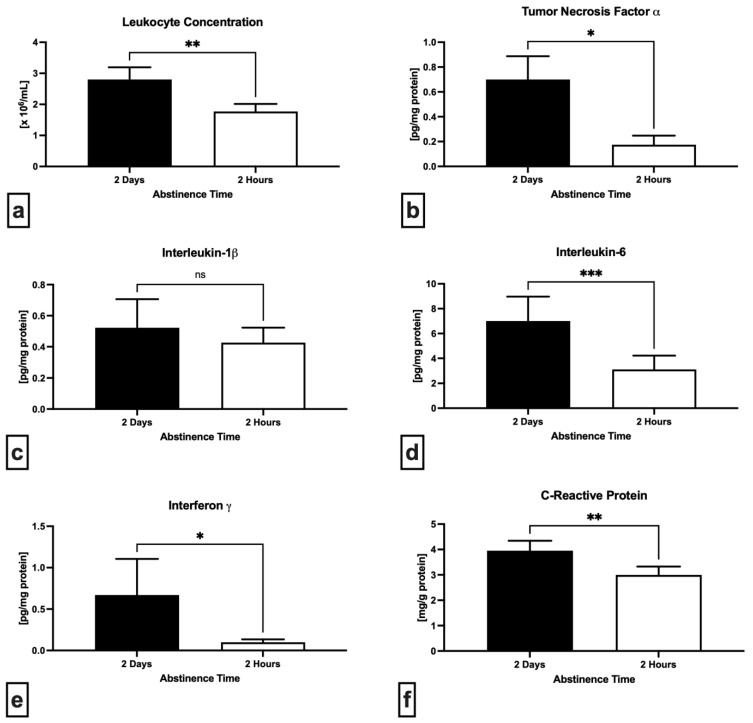
Variations in the concentration of leukocytes found in neat semen (**a**), levels of tumor necrosis factor alpha (**b**), interleukin-1β (**c**), interleukin-6 (**d**), interferon gamma (**e**) and C-reactive protein (**f**) in the seminal plasma following 2 days and 2 h of ejaculatory abstinence. * *p* < 0.05; ** *p* < 0.01; *** *p* < 0.001; ns—non-significant.

A notable improvement of the seminal immune profile was observed in the case of short abstinence (Figure 4). In comparison to 2 days of abstinence, specimens collected following 2 h exhibited a significantly lower leukocyte concentration (*p* = 0.0021; Figure 4a) as well as tumor necrosis factor alpha (TNF-α) levels (*p* = 0.0469; Figure 4b). While a non-significant decrease of interleukin-1 beta (IL-1β) was observed after a short abstinence (*p* = 0.9460; Figure 4c), interleukin-6 (IL-6) concentration decreased significantly in comparison to long abstinence (*p* = 0.0002; Figure 4d). Levels of both interferon gamma (IFN-γ) (Figure 4e) as well as C-reactive protein (CRP) (Figure 4f) were significantly decreased in semen samples obtained after 2 h in comparison to 2 days (*p* = 0.0156 with respect to IFN-γ; *p* = 0.0019 in case of CRP). 

### 2.4. Western Blot

**Figure 5:**
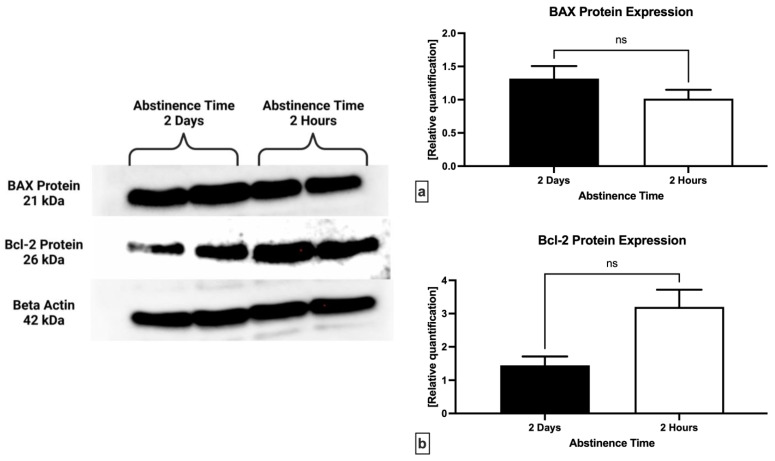
Expression patterns of BAX (**a**) and Bcl-2 protein (**b**) in semen samples collected following 2 days and 2 h of ejaculatory abstinence. ns—non-significant. Original photos of the blots are available as Supplementary Materials (Figure S1: Original blot of BAX protein; Figure S2: Inverted blot of BAX protein; Figure S3: Original blot of Bcl-2 protein; Figure S4: Inverted blot of Bcl-2 protein; Figure S5: Original blot of beta actin; Figure S6: Inverted blot of beta actin). Created with BioRender.com (accessed on 27 November 2022).

Relative quantification of the proteins involved in the regulation of cell death revealed a non-significant underexpression of the pro-apoptotic BAX protein in samples obtained following 2 h of abstinence when compared to 2 days (*p* = 0.3750; Figure 5a). Inversely, the anti-apoptotic Bcl-2 protein was found to be overexpressed following a short abstinence in comparison to long abstinence (*p* = 0.1250; Figure 5b), however without statistical significance. 

### 2.5. Bacteriological Analysis

**Figure 6:**
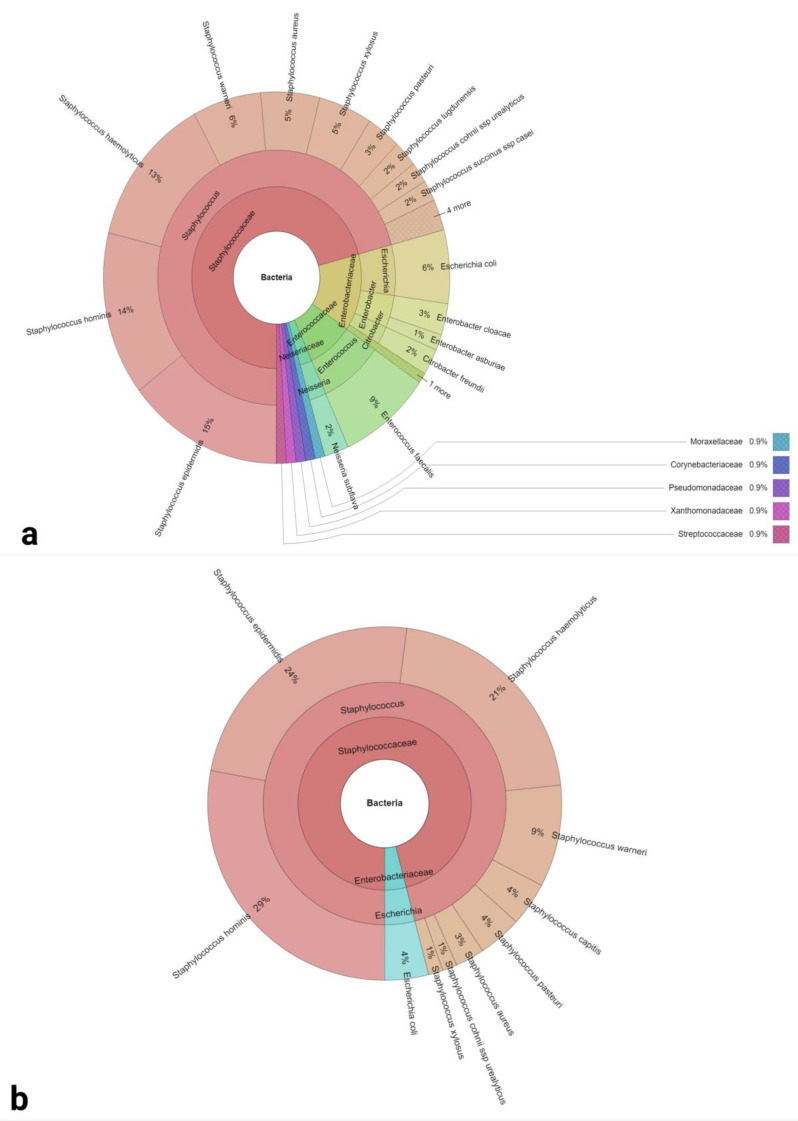
Krona charts of the bacteria recovered from semen samples collected following 2 days (**a**) and 2 h (**b**) of sexual abstinence (outermost ring: species, middle ring: genus, innermost ring: family).

Nine families, 12 genera and 27 bacterial species were detected by MALDI TOF mass spectrometry in semen samples collected following 2 days of ejaculatory abstinence (Figure 6a). Staphylococci were the predominant genus found in the samples (57%). Gram-positive bacteria were more frequent (65%) in comparison to Gram-negative bacteria (35%). Only staphylococci and *E. coli* were found in samples collected following 2 h of abstinence (Figure 6b). *Staphylococcus hominis* and *Staphylococcus epidermidis* were the predominant bacterial species (29% and 24%, respectively) in this case. 

Quantitatively, samples obtained after 2 h of abstinence presented with a significantly reduced bacterial load (*p* < 0.0001; Table 1). Whilst all semen samples collected following 2 days of abstinence tested positive for the presence of at least one bacterium, no bacteria were found in four samples obtained after 2 h of abstinence (Table 1).

### 2.6. Biodiversity Analysis

A total of 27 different bacterial species were found in samples collected following 2 days of abstinence, whereas 11 species were detected in the case of 2 h of abstinence. In both groups, *Staphylococcus epidermis* and *Staphylococcus hominis* were the most abundant bacterial species identified (Table 2).

Based on the obtained diversity indices, the richness of bacterial species was found to be higher following 2 days of ejaculatory abstinence. The calculated diversity indices were minimal in their values for both pre-established groups, which is strongly required in terms of reproductive biology. The Berger-Parker Index values were low in both groups as well as in what corresponds to small domination of individual bacterial species throughout the analyzed samples. Values of indexes were affected by a small abundance in numbers of bacteria present in the ejaculates (Table 3).

The comparative analysis carried out by ANOVA revealed significant differences among the groups for total counts and distribution of bacterial species that were identified in the samples (Table 4).

## 3. Discussion

The results of this study indicate that, similarly to previously published data [6,8,9,11,12,13,14,16,17], abstinence length has a positive impact on the semen volume and sperm concentration while at the same time being negatively correlated with other important semen quality characteristics such as sperm motility, viability, and DNA integrity. Despite statistically significant differences amongst the pre-established groups, real clinical implications were not apparent, since all key semen quality parameters were well within the reference values defined by the 6th edition of the WHO manual for the examination and processing of human semen, with stricter criteria concerning particularly the total sperm motility in comparison with the 5th edition (42% versus 40%, respectively) [40]. In this study, we established a very short ejaculatory abstinence period of 2 h, which, correspondingly to previous reports on extremely short abstinence lengths (i.e., less than 1–4 h), had a positive impact on conventional semen quality parameters [11,12,17,41] including sperm motion kinetics, DNA stability and plasma membrane integrity. Nevertheless, it must be acknowledged that data collected from similarly designed studies are not unanimous since Mayorga-Torres et al. [11] did not observe any correlation between abstinence duration and sperm motility, progressive motility, and vitality. Moreover, a decreasing trend was observed in these parameters at second, third, and fourth evaluations of semen collected following two hours of abstinence each when compared to the first assessment after 3–4 days of abstinence [16]. Another study revealed that although a second ejaculate collected 2 h following a first one had significantly higher total motile sperm count, no differences were observed in pregnancy rates after insemination [42]. It is generally acknowledged that increasing sperm concentration during prolonged abstinence periods affects the viscosity of semen, meaning that spermatozoa become closely packed within a restricted fluid space available for them to move freely, leading to alterations in their motion behavior [43]. Besides, with long ejaculatory abstinence, older and senescent spermatozoa progressively accumulate in the epididymis, contributing to a diminished semen quality and a higher rate of apoptotic cells [44,45] which is supported by our western blot analysis.

As in Ayad’s findings [17], the proportion of spontaneously acrosome-reacted spermatozoa was not found to have significant differences between short and long abstinence; nevertheless, a significant improvement in the mitochondrial membrane potential was observed in semen samples collected following 2 h of abstinence. This may provide additional support to the presence of higher motility rates after a short abstinence period, since mitochondrial function is crucial for a proper physiology, viability, and fertilization potential of male gametes [46]. A similar finding was published by Mayorga-Torres et al. [16], who observed stable and high ΔΨm levels throughout repeated semen collection at 2-h intervals, suggesting that the mitochondrial stability dependent on the sperm maturation process was not affected by recurrent ejaculation [44]. 

Beneficial or detrimental effects of ROS on the sperm structure and function are by and large dictated by the type of reactive intermediates involved in the physiological or pathological process as well as time of exposure which may be directly affected by ejaculatory abstinence [47]. In our case, global ROS levels were significantly decreased following 2 h in comparison to 2 days of abstinence. This may be explained by the fact that a shorter time of sperm storage in the epididymis decreases the risks of prolonged exposure to ROS produced by the aerobic metabolism of vital sperm cells or ROS released by damaged or dead spermatozoa [48]. While a decreasing trend in the levels of reactive intermediates following short abstinence time was observed earlier [13,16,17], this was not significant as it was in our case. It may be noted, however, that previous reports referred to short ejaculatory abstinence within a timeframe of one day rather than hours [13,16] or were focused on one specific reactive intermediate that either may not necessarily cause excessive oxidative damage, or which may be easily scavenged by the antioxidant system present in the seminal plasma [17]. Correspondingly, lower levels of oxidative damage to the sperm proteins and lipids were observed in semen samples collected following 2 h of abstinence, indicating a lower risk of damage to biomolecules vital for the sperm survival because of reduced ROS levels and a decreased probability for oxidative chain reactions to occur. Interestingly, the antioxidant capacity of the seminal plasma was slightly reduced following short ejaculatory abstinence, which contradicts Mayorga-Torres [16] and Ayad [17] who observed an improvement the antioxidant status of seminal plasma, particularly in the activity of superoxide dismutase and catalase. We may speculate that the decrease of TAC may be related to the reduced semen volume following short abstinence time. It is well known that the seminal plasma acts as a reservoir of molecules that are either inherently designed to counteract ROS or to sustain an environment favoring ROS scavenging and neutralization [49]. Since the semen samples collected following 2 h of abstinence had a lower volume, it may be feasible to expect a decreased volume of the seminal plasma, and thus a diminished quantity of primary and secondary antioxidants. Also, since antioxidants are designed to counteract ROS and thus maintain an optimal oxidative balance, the decrease of TAC may mirror a reduced ROS concentration found in the ejaculates. 

Currently available data on the effects of ejaculatory abstinence on DNA integrity are incoherent and often contradictory, primarily due to different sample sizes or studied populations as well as methods used for the assessment of the sperm DNA fragmentation index. While Mayorga-Torres et al. [11] observed no significant changes in the proportion of spermatozoa with alterations to the DNA molecule, an increasing trend was observed in sperm DNA fragmentation with each recurrent semen collection at 2-h intervals after the first one, although no specimen trespassed acceptable levels (<29%) established for sperm DNA damage [50]. According to De Jonge et al. [14], frequent ejaculation may increase the occurrence of immature spermatozoa with defects in the chromatin packaging. In contrast, our data agree with Gosalvez et al. [12], who observed a reduced incidence of sperm DNA fragmentation after short-term recurrent ejaculations. Similar studies have shown that shorter abstinence timeframes lead to a reduction in the incidence of sperm DNA damage followed by increased pregnancy rates after ARTs [12,18]. According to Chen et al. [51], an inverse correlation between abstinence time and sperm DNA fragmentation may be attributed to a shorter exposure of spermatozoa to ROS during the epididymal transit and storage. Furthermore, with the increase of abstinence time, excessive sperm apoptosis caused by internal or external stimuli may also contribute to an increased DNA fragmentation [52].

As discussed earlier, relationships amongst bacteria present in ejaculates and male gametes have just recently begun to be unraveled. Moreover, data on normozoospermic males are sparse, since the impact of bacteriocenoses on the sperm structural integrity and functional activity have been by and large studied in males objectively suffering from urogenital infections, sub- or infertility [53,54,55]. In our case, all samples collected following 2 days and 75% of specimens obtained after 2 h of abstinence tested positive for the presence of bacteria. This is not an unusual phenomenon, since it has been previously established that up to 70–85% of ejaculates obtained from otherwise healthy and fertile subjects may be contaminated with bacteria [21,22,56,57]. As indicated in an earlier report, the severity of effects bacteria may exhibit on male gametes, are directly dependent upon the bacterial diversity and the overall quantity of bacteria present in the sample, also known as bacterial load [22]. In terms of the bacterial load, a significant decrease of this parameter was observed following short abstinence. The reason for this difference may be explained by a shorter timeframe for the growth and accumulation of bacteria within the male urogenital tract, which may be applicable to the sperm concentration as well. Whilst it has been observed that urination helps to flush out bacteria that are regular commensals of the male reproductive tract, we may exclude this factor, since all participants were asked to urinate prior to each collection. Significant changes were also observed in the bacterial diversity. Samples obtained following 2 days of collection presented with a greater variability of bacterial genera and species, most of which were G^+^ commensal staphylococci as in earlier bacteriological studies [22,56,58]. Nevertheless, it must be acknowledged that *E. coli*, *S. aureus*, *S. haemolyticus* and *P. aeruginosa*, considered to be potential uropathogens, were detected in this study, agreeing with previous reports on normozoospermic men [21,22,57]. The origin of these bacterial species is subject to debate. Whilst the participants were instructed to follow strict hygiene standards prior and during semen collection, a potential contamination of the samples stemming from the hands, foreskin or collection supplies cannot be excluded and should be verified. Since all samples passed the inclusion criteria set by the study, we may speculate, that the quantity of uropathogens did not reach the threshold to exhibit signs indicative of clinical bacteriospermia. In the meantime, 16 out of 26 bacterial species were not detected in specimens collected following 2 h in comparison to 2 days. Interestingly, only *E. coli* and several staphylococci were identified following short abstinence, changing the resulting diversity of seminal bacteriocenoses. Since this phenomenon has not been previously studied, we may only hold on to speculations suggesting the existence of specific features that enable *E. coli* and staphylococci to be more persistent in adherence to host cells in comparison to other bacterial genera. These may be represented by specific adhesive molecules found on the bacterial cell wall and/or membrane. 

According to Zhang et al. [59], bacterial adhesion to the sperm surface may result in an increased load of cells and thus impair the membrane integrity and overall vitality of spermatozoa. Bacteria immobilized by adherence may subsequently attract other bacteria that will form agglutinating complexes, leading to alterations in the sperm motion [60]. Subsequent sperm-bacterial agglutination may trigger the release of extracellular polymeric substances and initiate biofilm formation [61,62]. At the same time, a successfully accomplished adhesion process may favor the release of bacterial metabolites with cytotoxic effects on the male gamete [63]. *E. coli* are known to contain polymeric structures called “fimbriae” or “pilli” that serve to establish contact with and attachment to the host cell. Specifically, Type 1 fimbrinae considered to be the most versatile virulence factor of uropathogenic *E. coli* has high affinity to mannose receptors [60] present on the sperm head [64]. In the meantime, P fimbriae often found in *E. coli* strains during acute prostatitis [62] recognizes a-D-galp-l-4-9-D-galp (gal gal) located primarily in the sperm tail [65]. In the case of G^+^ bacteria, particularly staphylococci, a group of proteins called microbial surface components recognize adhesive matrix molecules target proteins in the host’s extracellular matrix [59] and induce a strong affinity to selected hydrophobic molecules once the adhesion process is initiated [66]. A particular colonization advantage in staphylococci is provided by the so-called moonlight proteins that act as particularly resilient adhesins [59,67]. All in all, it may be hypothesized that the above-mentioned molecules may provide a higher resilience and persistence of *E. coli* and staphylococci in the male reproductive tract and fluids, leading to their recurrence following repeated ejaculation. 

Changes to the sperm quality characteristics, including motility, mitochondrial activity, or membrane integrity in the presence of an increased bacterial load have been already reported to occur not only in sub- or infertile patients [53,55,68,69,70] but also in normozoospermic males [21,22,56,57]. Negative impact of bacteria on the sperm architecture and function has been furthermore observed in preliminary in vitro studies [37,38,58], fortifying the hypothesis that bacteriocenoses may at least partially influence the resulting semen quality. As such, it may be plausible to assume that variations in the bacterial profiles could represent another factor to be considered with respect to changes in the semen quality following a long or short ejaculatory abstinence.

Besides sperm senescence, bacteria could be involved in a higher expression of the pro-apoptotic BAX protein since a greater proportion of spermatozoa expressing early and/or late apoptotic markers was observed following exposure to pathogenic or conditionally pathogenic bacterial species [71,72]. What is more, sperm cell death by apoptosis or necrosis was triggered already by a simple contact with bacteria without the involvement of inflammatory events [21,71]. Our collected data agree with these observations, since we detected a decline of ΔΨm that was accompanied by elevated sperm DNA damage and loss of membrane integrity in the samples collected following two days of abstinence that contained higher bacterial loads and occurrences of typical uropathogenic bacteria. 

Inherently, the immune system responds to infection by releasing white blood cells to the source of inflammation. While within the male reproductive system leukocytes are crucial for the removal of abnormal and/or dead spermatozoa, their overactivation triggered by a tight adherence to male gametes may lead to phagocytosis of even structurally and functionally viable spermatozoa [73,74,75]. It has been previously observed that longer ejaculatory abstinence as well as the presence of notably coliform bacteria may lead to a higher incidence of leukocytes in semen with subsequent damage particularly to the membranous structures of male gametes. This observation corresponds to earlier studies [21,22], observing that bacteria alongside leukocytes compromised the lipid symmetry of the sperm membranes even in young normozoospermic men. 

An accompanying phenomenon of active immune response lies in the release of pro-inflammatory cytokines, which may act as spermatotoxins. Besides promoting oxidative damage primarily to the sperm membranes via lipid peroxidation [76], it has been postulated that these molecules participate in the induction of cell death. Within the vast array of pro-inflammatory cytokines, TNF-α and IFN-γ, which are the predominant molecules secreted during infection and/or inflammation, may trigger sperm phosphatidylserine translocation or DNA fragmentation [77,78]. Among interleukins, IL-1 and IL-6 also seem to mediate damage to male gametes, which agrees with our observations on their increasing levels in proportion to a diminished semen quality following a longer abstinence interval. Accordingly, their increased levels as a consequence of higher bacterial load have been associated with a decreased sperm quality even in normozoospermic males [22,79,80]. What is more, cytokines have been previously linked to seminal oxidative stress [76,81] and a compromised sperm motility [38,82], all of which was revealed by our results as well. A possible explanation to these phenomena may be connected to the sperm storage time in the epididymis. During prolonged abstinence periods, spermatozoa passing through the cauda epididymis are permanently exposed to different quiescence factors [83,84], which may negatively affect their future vitality following ejaculation. If bacteria are present during sperm maturation and storage, spermatozoa will have to withstand a less favorable environment caused by bacterial action as well as by the cells and molecules of the immune system. As such, the release of spermatozoa through more frequent ejaculations may diminish the action of such inhibitory factors, as their levels will decrease to basal values. This may ultimately help to reduce the severity of such factors and minimize their potential detrimental effects on the sperm structural integrity and functional activity.

Besides inducing inflammation, oxidative stress plays an important role in mediating damage to male gametes. Spermatozoa, leukocytes, aerobic as well as facultative anaerobic bacteria release ROS as their metabolic by-products. Even anaerobes can deploy low-potential electron transfer pathways to synthesize reactive intermediates [48]. ROS have been shown to be produced by a variety of potentially uropathogenic bacteria, such as *E. coli* [85], *S. aureus* [86], and *E. faecalis* [87], elevated loads of which may contribute to the progression of oxidative damage to spermatozoa. Besides higher ROS levels, our data unravel a notable rise in protein carbonyls and MDA in samples collected following longer ejaculatory abstinence, which also contained a higher bacterial load. Supraphysiological ROS may attack the lipid bilayer of sperm membranes, which will have an undesirable impact on the semipermeable properties of the sperm surface. Our findings may furthermore support the hypothesis that apoptosis could play important roles in promoting ROS-inflicted sperm DNA damage [21,22]. Accordingly, an increase in the proportion of spermatozoa with fragmented DNA correlated with an elevated BAX/Bcl-2 ratio in semen obtained following longer ejaculatory abstinence with more uropathogenic bacteria, which has also been reported in infertile men suffering from urogenital infections [38,88,89].

Finally, our data suggest that ejaculatory abstinence affects the seminal microbiome which may contribute to changes in the semen quality following a long or short abstinence period. It seems that *E. coli* and staphylococci are more resilient to frequent ejaculation and remain present in semen even after a short ejaculatory abstinence. In this sense, a series of short-term repeated semen collections could shed more light on a time-dependent recurrence of these bacteria in subsequent semen specimens. At the same time, more comprehensive bacteriological studies could elucidate specific characteristics that enable *E. coli* and staphylococci to persistently re-occur during repeated ejaculation. One factor limiting us in drawing more substantial conclusions lies in the fact that samples obtained from normozoospermic males were used in this study. Bacterial profiles of semen are known to change among individuals and may be affected by numerous intrinsic and extrinsic factors. As such, it would be of value to study ejaculatory abstinence-related changes to the seminal microbiome in subjects suffering from sub- or infertility. If indeed sexual abstinence decreases the bacterial load or the presence of potentially uropathogenic species in ejaculates, and if enough high-quality spermatozoa are retrieved for ARTs, sperm processing and culture media could be supplemented with narrow-spectrum antibiotics, thus preventing unnecessary overuse of broad-spectrum antibiotics during semen handling and associated risks for the spread of antibiotic-resistant bacteria. Nevertheless, these strategies rely on the development of fast, straightforward and cost-effective bacteriological screening methods for practical andrology.

## 4. Materials and Methods

### 4.1. Collection and Processing of Ejaculates

Fifty-one healthy volunteers aged 21–38 years were recruited for this study. The established criteria for sample inclusion were as follows: (1) normal semen quality according to the sixth edition of the WHO Manual for the Laboratory Examination and Processing of Human Semen [5]; (2) no history of reproductive dysfunction; and (3) no current or previous urogenital infection. All volunteers were informed about the aim and expected outcomes of the study and subsequently signed informed consents. All procedures complied with the 1964 Helsinki Declaration and its later amendments or comparable ethical standards.

Prior to semen collection the participants were instructed to urinate, wash their hands and external genitalia with soap and water, and dry them off with disposable paper towels. Two semen samples were collected from each participant by masturbation. The first specimen was obtained following 2 days of sexual abstinence. This was followed by a second semen collection after 2 h of abstinence. All semen samples were collected into sterile containers and subsequently allowed to liquefy for 30 min at 37 °C.

Following liquefaction and volume measurement, each ejaculate was divided into four aliquots. The first aliquot was immediately subjected to the assessment of sperm motility, membrane, acrosome and DNA integrity, ROS production and leukocyte concentration. The second aliquot was transferred to an Eppendorf tube and stored at −80 °C for bacteriological analysis. The third aliquot was centrifuged at 300× *g* for 10 min to obtain seminal plasma which was subjected to the assessment of protein concentration and subsequently stored at −80 °C for the evaluation of total antioxidant capacity (TAC) and ELISA assays. The final aliquot of native semen was processed with a single layer gradient separation using 80% Percoll^®^ (Sigma-Aldrich, St. Louis, MO, USA) with HEPES-buffered Ham’s F-10 medium (Sigma-Aldrich, St. Louis, MO, USA), according to the protocol established by Sharma et al. [90]. Obtained spermatozoa were centrifuged (300× *g*, 7 min) and washed with Dulbecco’s Phosphate Buffered Saline (DPBS; without calcium chloride and magnesium chloride; Sigma-Aldrich, St. Louis, MO, USA) three times. The samples were then solubilized in RIPA lysis buffer (Merck, Darmstadt, Germany) containing a proteinase inhibitor cocktail (Sigma-Aldrich, St. Louis, MO, USA) overnight at 4°C to allow a complete sperm lysis. After centrifugation at 13,000× *g* for 30 min, the supernatant was aspirated, subjected to the determination of the protein concentration, and stored at −80 °C for later assessment of oxidative damage to the proteins and lipids as well as for Western blot analysis [91].

Protein concentration in the seminal plasma and cell lysates was determined using the Total Protein assay commercial kit (Randox, Crumlin, UK) and RX Monaco fully automated clinical chemistry analyzer (Randox, Crumlin, UK) [86]. 

### 4.2. Semen Quality Analysis

The HTM TOX IVOS II. Computer-assisted semen analysis (CASA) system (version 14.0; Hamilton-Thorne Biosciences, Beverly, MA, USA) was used to assess the sperm motility defined as the percentage of total motile spermatozoa (%). Each semen sample was diluted with DPBS, stained with the IDENT stain (final concentration of 10 μg/mL; Hamilton-Thorne Biosciences, Beverly, MA, USA) and evaluated under fluorescent illumination as previously described by Tvrdá et al. [22]. 

A dual staining protocol employing carboxyfluorescein diacetate (CFDA) and DAPI (4′,6-diamidino-2-phenylindole) was used to assess the membrane integrity. Semen samples diluted with pre-warmed DPBS to 10^6^ cells were stained with 10 μL CFDA (0.75 mg/mL in dimethyl sulfoxide; Sigma-Aldrich, St. Louis, MO, USA) and 10 μL DAPI (1 μmol/L in DPBS; Sigma-Aldrich, St. Louis, MO, USA) and incubated at 37 °C in the dark for 15 min. Each sample was then centrifuged (150× *g*, 5 min, 20 °C) and washed with 100 µL DPBS twice. Finally, the samples were resuspended in 100 µL PBS, and a minimum of 300 cells were evaluated using an epifluorescence microscope (×40) (Leica Microsystems, Wetzlar, Germany). CFDA-positive cells were classified as membrane-intact and expressed in percentage (%) [92,93]. 

For the acrosome integrity, 10^6^ spermatozoa were stained with 100 μL PNA (peanut agglutinin, FITC conjugate; Sigma-Aldrich, St. Louis, MO, USA; 10 μM in PBS) and 10 μL DAPI. Following incubation at 37 °C for 30 min, at least 300 cells were counted under an epifluorescence microscope (×40). PNA-negative spermatozoa were classified as acrosome-intact and expressed in percentage (%) [22].

Mitochondrial membrane potential (ΔΨm) was assessed with the commercially available JC-1 kit (Cayman Chemical, Ann Arbor, MI, USA). Suspensions containing 10^6^ spermatozoa were stained with 5 μL JC-1 working solution. Following incubation at 37 °C for 20 min, the samples were centrifuged at 150× *g*, 20 °C for 10 min, washed twice with a washing buffer provided by the kit, transferred to a black 96-well plate, and the proportion of JC-1 monomers and polymers was quantified with the Glomax Multi^+^ combined spectro-fluoro-luminometer (Promega Corporation, Madison, WI, USA). Mitochondrial activity is expressed as the ratio of JC-1 monomers to JC-1 complexes (green/red ratio) [22].

Sperm DNA integrity was assessed with the Halosperm^®^ commercial kit (Halotech DNA, Madrid, Spain). Briefly, semen samples were mixed with low-melting point agarose, transferred onto agarose-covered microscopic slides, and exposed to an acid solution (7 min), followed by a lysis solution (20 min). Following washing with distilled water and dehydration in 70% and 100% ethanol, air-dried slides were stained with SYBR Green (2 μg/mL; Sigma Aldrich, St. Louis, MO, USA). In the absence of massive DNA breakage, spermatozoa produced nucleoids with large halos of spreading DNA loops, emerging from a central core. On the other hand, nucleoids emerging from spermatozoa with fragmented DNA either did not show a dispersion halo or the halo was minimal. At least 300 spermatozoa were scored under an epifluorescence microscope (×40) and the proportion of spermatozoa with undamaged DNA was expressed in % [94].

The Endtz test was used to quantify the number of leukocytes in each specimen. Each ejaculate was treated with the Endtz solution containing 96% ethanol (Centralchem, Bratislava, Slovakia), benzidine (Sigma-Aldrich, St. Louis, MO, USA), sterile water, and 3% hydrogen peroxide (H_2_O_2_; Sigma-Aldrich, St. Louis, MO, USA). Following incubation (20 °C, 5 min), stained round cells were counted with the Nikon ECLIPSE E100 bright-field micro-scope (Nikon, Tokyo, Japan; ×1000). The results are expressed as ×10^6^ leukocytes/mL semen [22].

### 4.3. Oxidative Profile

Luminol-based chemiluminescent assay was employed to quantify the extent of ROS production in the semen samples. Briefly, each ejaculate was transferred to a 96-well plate and mixed with 5 mM luminol (5-Amino-2,3-dihydro-1,4-phthalazinedione; Sigma-Aldrich, St. Louis, MO, USA). Negative controls consisted of DPBS and luminol, while positive controls comprised DPBS, H_2_O_2_ (33%; Sigma-Aldrich, St. Louis, MO, USA) and luminol. The light signal emitted from the reaction was monitored with the Glomax Multi^+^ combined spectro-fluoro-luminometer (Promega Corporation, Madison, WI, USA). The results are expressed in relative light units per second per one million sperm (RLU/s/10^6^ sperm) [22].

A chemiluminescent assay designed by Muller et al. [95] was used to study the TAC of the seminal plasma. Each sample was treated with a signal reagent comprised of luminol, 4-iodophenol (Sigma-Aldrich; St. Louis, MO, USA), horseradish peroxidase (HRP; Sigma-Aldrich; St. Louis, MO, USA), and H_2_O_2_. Chemiluminescence was observed during 10 consecutive cycles of 1 min with the Glomax Multi^+^ combined spectro-fluoro-luminometer (Promega Corporation, Madison, WI, USA). The collected data were processed with the help of a Trolox (5–100 μmol/L; 6-hydroxy-2,5,7,8-tetramethylchroman-2-carboxylic acid; Sigma-Aldrich; St. Louis, MO, USA) standard curve. The results are expressed as μmol Trolox Eq./g protein [22].

Oxidative damage to the sperm proteins expressed through the amount of protein carbonyls (PC) in the sperm lysates was assessed with a modified DNPH (dinitrophenylhydrazine) assay [96]. Each lysate was adjusted with distilled water to 1 mg protein/1 mL, pre-treated with 1 mL of trichloroacetic acid (TCA; Sigma-Aldrich, St. Louis, MO, USA) and incubated for 10 min at 4 °C. Following centrifugation (805× *g*, 10 min, 4 °C), the resulting pellet was exposed to 1 mL DNPH (Sigma-Aldrich, St. Louis, MO, USA) and incubated at 37 °C for 60 min. Subsequently, 1 mL TCA was added to the mixture, the samples were cooled down and centrifuged again (805× *g*, 5 min, 4 °C). The pellet was washed 3 times with 500 µL ethyl acetate/ethanol solution (50/50 mix; Sigma-Aldrich, St. Louis, MO, USA). Finally, the resulting pellet was resuspended in 1 mL 6 M guanidine hydrochloride (Sigma-Aldrich, St. Louis, MO, USA) and the resulting absorbance was measured at 360 nm using a UV-VIS spectrophotometer (Cary Systems, Santa Clara, CA, USA). Oxidative damage to the proteins is expressed in nmol PC/mg protein [97].

Thiobarbituric acid-reactive substances (TBARS) assay was employed to assess the extent of lipid peroxidation expressed through malondialdehyde (MDA) amounts. Briefly, the lysates were pre-treated with 5% SDS (sodium dodecyl sulfate; Sigma-Aldrich, St. Louis, MO, USA) and 0.53% thiobarbituric acid (Sigma-Aldrich, St. Louis, MO, USA), dissolved in 20% acetic acid (pH 3.5; Centralchem, Slovakia). The mixture was boiled (100 °C) for 1 h, subsequently cooled down for 10 min and centrifuged (1300× *g*, 10 min, 4 °C). The supernatants were transferred to a 96-well plate, and the final absorbances were measured with the Glomax plate spectrophotometer (Promega Corporation, Madison, WI, USA) at 540 nm. MDA levels were calculated with the help of a standardization curve constructed from pre-prepared MDA standards. The extent of lipid peroxidation is expressed in µmol MDA/mg protein [98].

### 4.4. ELISA Assays

Commercially available ELISA kits were purchased from Invitrogen (Waltham, MA, USA) for the quantification of selected pro-inflammatory factors including TNF-α (#BMS223-4), IL-1β (#KAC1211), IL-6 (#EH2IL6), IFN-γ (#BMS228) and CRP (#KHA0031). A double-sandwich ELISA protocol was performed according to the instructions of the manufacturer, and the absorbances were read with the help of the Glomax plate spectrophotometer (Promega, Madison, WI, USA) at 450 nm.

### 4.5. Western Blots

Two randomly selected pairs of samples with a suitable protein concentration were selected for the Western blot analysis of the pro-apoptotic BAX protein and the anti-apoptotic Bcl-2 protein. Prior to the assay, the protein concentration of the samples was adjusted using ultrapure (UHQ) water to reach a final concentration of 25 μg protein. The samples were then treated with 4× Laemli buffer (BioRad, Hercules, CA, USA) and β-mercaptoethanol (Sigma-Aldrich, St. Louis, MO, USA), and subsequently boiled at 95 °C for 10 min. The samples were loaded (20 μL) into Mini-PROTEAN TGX stain-free polyacrylamide gels (BioRad, Hercules, CA, USA), together with 7 μL of Precision Plus Protein marker (BioRad, Hercules, CA, USA). SDS-polyacrylamide gel electrophoresis was run at 90 V for 2 h, and the gels were visualized with the ChemiDoc Imaging System (BioRad, Hercules, CA, USA) to confirm the loading uniformity. The gels were then transferred to polyvinylidene difluoride membranes (Trans-Blot Turbo Pack; BioRad, Hercules, CA, USA) using the Trans-Blot Turbo Transfer System (BioRad, Hercules, CA, USA) at 25 V and 2.5 A, for 7 min. The blotting sandwich was disassembled, and the membranes were incubated for 3 × 10 min in Tris-buffered saline (TBS) composed of Tris base (Sigma-Aldrich, St. Louis, MO, USA), sodium chloride (Centralchem, Bratislava, Slovakia), and UHQ water. This step was followed by membrane staining with Ponceau S solution (Sigma-Aldrich, St. Louis, MO, USA) to visualize the bands on the membranes. Subsequently, the membranes were cut into smaller pieces, where the protein of interest was presumably localized. The membranes were blocked with 5% skim milk (Blotting grade blocker; BioRad, Hercules, CA, USA) in TBS containing 0.1% Tween-20 (Sigma-Aldrich, St. Louis, MO, USA). Membrane blocking was performed on a stirrer at room temperature for 2 h. Finally, the membranes were incubated with one of the following primary antibodies:

- anti-BAX antibody (BCL2-Associated X Protein) N-Term (#ABIN6990475; Antibodies Online; Dunwoody, GA, USA); Polyclonal/IgG; source: rabbit; 1:1000 in 5% milk in TBS/0.1% Tween-20.
- anti-Bcl-2 antibody (B-Cell CLL/lymphoma 2) N-Term (#ABIN2857047; Antibodies Online; Dunwoody, GA, USA); Polyclonal/IgG; source: rabbit; 1:1000 in 5% milk in TBS/0.1% Tween-20.
- β-actin antibody (AC-15) (#AM4302; Invitrogen, Waltham, MA, USA); Monoclonal/IgG; source: mouse; 1:1000 in 5% milk in TBS/0.1% Tween-20.

The next day, the membranes were washed for 5 × 10 min in a wash buffer composed of 1% milk in TBS/0.2% Tween-20, and subsequently incubated with a secondary anti-rabbit antibody (for BAX and Bcl-2) (GE Healthcare, Chicago, IL, USA) or anti-mouse antibody (for β-actin) diluted 1:15 000 in 1% milk in TBS/0.2% Tween-20 for 1 h. Following incubation, the membranes were washed for 3 × 10 min in TBS/0.2% Tween-20 at room temperature and using a stirrer. To visualize the target protein, membranes were incubated with the ECL substrate (GE Healthcare, Chicago, IL, USA) in the dark for 5 min. After incubation, the membranes were placed into the ChemiDoc Imaging System, which automatically calculated the protein visualization time based on the light signal emitted by the membranes [99].

### 4.6. Bacterial Cultures and Identification 

To assess changes in the bacterial profiles following 2 days and 2 h of abstinence, 100 µL of each specimen was inoculated on a selection of sterile agars (blood agar base no. 2, malt extract agar, trypticase soy agar; Merck, Darmstadt, Germany) and incubated under aerobic conditions at 36 ± 2 °C for 24 h. Grown bacterial colonies were counted and re-inoculated and incubated again under aerobic conditions at 37 ± 1 °C for 24 h [22].

Pure bacterial colonies were identified with the matrix assisted laser desorption/ionization time-of-flight (MALDI-TOF) Biotyper mass spectrometry (Brucker Daltonics, Bremen, Germany), previously described by Tvrdá et al. [22]. Briefly, purified cultures re-suspended in distilled water were treated with 99.8% ethanol (Sigma-Aldrich, St. Louis, MO, USA) and air-dried. The pellet was mixed thoroughly with a solution comprising acetonitrile (Sigma-Aldrich, St. Louis, MO, USA), 70% formic acid (Sigma-Aldrich, St. Louis, MO, USA), centrifuged and transferred to the MALDI identification plate. Dried specimens were covered with a working solution of MALDI matrix and assessed with the Microflex LT instrument and the flexControl software (version 3.4). Obtained data were processed with the MALDI Biotyper Bruker Taxonomy database (Bruker Daltonics, Bremen, Germany) [22].

### 4.7. Biodiversity Calculation

The total number of species obtained in the pre-established groups were determined as species richness. Standard basic diversity parameters were calculated by the BPMSG Diversity Calculator. Besides standard indices, Berger-Parker Index was calculated based on the formula **d = max(pi)** to characterize real unbalanced group differences in the pre-established groups. ANOVA was calculated for the groups by the astatsa.com free software platform. 

### 4.8. Statistical Analysis

GraphPad Prism (version 9.4.1 for Mac; GraphPad Software Incorporated, La Jolla, CA, USA) was used for the statistical analysis. The results are expressed as mean (±SD). All data were subjected to the Shapiro-Wilk normality test. Differences between the paired samples were analyzed by the Wilcoxon matched pairs signed rank test. Statistical significance was set at * *p* < 0.05; ** *p* < 0.01; *** *p* < 0.001; **** *p* < 0.0001.

## Figures and Tables

**Table 1:** Bacterial profiles and sample positivity of semen specimens collected following 2 days or 2 h of abstinence.

Groups	2 Days of Abstinence	2 h of Abstinence
Bacterial Load (log_10_ CFU/mL)	3.27 ± 0.34	1.59 ± 0.35 ****
Identified Bacterial Species and Sample Positivity	*Staphylococcus epidermidis* (62.75%)	*Staphylococcus epidermidis* (35.29%)
	*Staphylococcus hominis* (60.78%)	*Staphylococcus hominis* (41.18%)
	*Staphylococcus haemolyticus* (56.86%)	*Staphylococcus haemolyticus* (29.41%)
	*Staphylococcus warneri* (27.45%)	*Staphylococcus warneri* (13.73%)
	*Escherichia coli* (25.49%)	*Escherichia coli* (5.88%)
	*Staphylococcus xylosus* (21.56%)	*Staphylococcus xylosus* (1.96%)
	*Staphylococcus cohnii* (15.69%)	*Staphylococcus cohnii* (3.92%)
	*Staphylococcus pasteuri* (13.73%)	*Staphylococcus pasteuri* (5.88%)
	*Staphylococcus capitis* (13.73%)	*Staphylococcus capitis* (5.88%)
	*Staphylococcus aureus* (11.76%)	*Staphylococcus aureus* (3.92%)
	*Enterococcus faecalis* (37.25%)	N/A
	*Enterobacter cloacae* (11.76%)	N/A
	*Citrobacter freundii* (9.80%)	N/A
	*Neisseria subflava* (9.80%)	N/A
	*Staphylococcus lugdunensis* (9.80%)	N/A
	*Staphylococcus succinus* (7.84%)	N/A
	*Enterobacter asburiae* (5.88%)	N/A
	*Acinetobacter lwoffii* (3.92%)	N/A
	*Corynebacterium glucuronolyticum* (3.92%)	N/A
	*Pseudomonas aeruginosa* (3.92%)	N/A
	*Rahnella aquatilis* (3.92%)	N/A
	*Staphylococcus chromogenes* (1.96%)	N/A
	*Staphylococcus schleiferi* (3.92%)	N/A
	*Staphylococcus simiae* (3.92%)	N/A
	*Stenotrophomonas maltophilia* (3.92%)	N/A
	*Streptococcus agalactiae* (3.92%)	N/A

**** *p* < 0.0001.

**Table 2:** Bacterial species identified in the pre-established groups.

Species	2 Days of Abstinence	2 h of Abstinence
*Staphylococcus epidermidis*	32	18
*Staphylococcus hominis*	31	21
*Staphylococcus haemolyticus*	29	16
*Enterococcus faecalis*	19	-
*Escherichia coli*	14	3
*Staphylococcus warneri*	14	7
*Staphylococcus xylosus*	11	1
*Staphylococcus pasteuri*	7	3
*Staphylococcus aureus*	6	2
*Enterobacter cloacae*	6	-
*Staphylococcus capitis*	6	3
*Staphylococcus lugdunensis*	5	-
*Neisseria subflava*	5	-
*Citrobacter freundii*	5	-
*Staphylococcus cohnii* ssp. *Urealyticus*	4	1
*Staphylococcus succinus* ssp. *casei*	4	-
*Enterobacter asburiae*	3	-
*Corynebacterium glucuronolyticum*	2	-
*Pseudomonas aeruginosa*	2	-
*Streptococcus agalactiae*	2	-
*Staphylococcus simiae*	2	-
*Stenotrophomonas maltophilia*	2	-
*Staphylococcus schleiferi*	2	-
*Rahnella aquatilis*	2	-
*Acinetobacter lwoffii*	2	-
*Staphylococcus chromogenes*	1	-
*Staphylococcus capitis* ssp. *capitis*	1	-
N/A	-	4

**Table 3:** Bacterial biodiversity characteristics of the pre-established groups.

Groups	2 Days of Abstinence	2 h of Abstinence
Richness (R)	27	11
Berger Parker Dominance Index	0.16	0.27
Shannon α-diversity	2.54	1.96
Simpson dominance	0.1	0.18

**Table 4:** ANOVA analysis of the pre-established groups.

Source	Sum of Squares (SS)	Degrees of Freedom (ν)	Mean Square (MS)	F Statistic	*p*-Value
**Treatment**	7.69	1	7.69	80.7043	1.1102 × 10^−16^
**Error**	252.8235	2751	0.0919		
**Total**	260.2405	2752			

## Data Availability

The data presented in this study are available on request from the corresponding author.

## Supplementary Materials

The following supporting information can be downloaded at: https://www.mdpi.com/article/10.3390/ijms24043503/s1.

## References

[1] Mann U., Shiff B., Patel P. (2020). Reasons for worldwide decline in male fertility. Curr. Opin. Urol..

[2] Jafari H., Mirzaiinajmabadi K., Roudsari R.L., Rakhshkhorshid M. (2021). The factors affecting male infertility: A systematic review. Int. J. Reprod. Biomed..

[3] Baskaran S., Finelli R., Agarwal A., Henkel R. (2021). Diagnostic value of routine semen analysis in clinical andrology. Andrologia.

[4] Milachich T., Dyulgerova-Nikolova D., Sharma N., Chakrabarti S., Barak Y., Ellenbogen A. (2020). The Sperm: Parameters and Evaluation. Innovations in Assisted Reproduction Technology.

[5] World Health Organization (2021). WHO Laboratory Manual for the Examination and Processing of Human Semen.

[6] Cooper T.G., Keck C., Oberdieck U., Nieschlag E. (1993). Effects of multiple ejaculations after extended periods of sexual abstinence on total, motile and normal sperm numbers, as well as accessory gland secretions, from healthy normal and oligozoospermic men. Hum. Reprod..

[7] Pellestor F., Girardet A., Andreo B. (1994). Effect of long abstinence periods on human sperm quality. Int. J. Fertil. Menopausal. Stud..

[8] Okada F.K., Andretta R.R., Spaine D.M. (2020). One day is better than four days of ejaculatory abstinence for sperm function. Reprod. Fertil..

[9] Levitas E., Lunenfeld E., Weiss N., Friger M., Har-Vardi I., Koifman A., Potashnik G. (2005). Relationship between the duration of sexual abstinence and semen quality: Analysis of 9,489 semen samples. Fertil. Steril..

[10] Dutta S., Majzoub A., Agarwal A. (2019). Oxidative stress and sperm function: A systematic review on evaluation and management. Arab. J. Urol..

[11] Mayorga-Torres J.M., Agarwal A., Roychoudhury S., Cadavid A., Cardona-Maya W.D. (2016). Can a Short Term of Repeated Ejaculations Affect Seminal Parameters?. J. Reprod. Infertil..

[12] Gosálvez J., González-Martínez M., López-Fernández C., Fernández J.L., Sánchez-Martín P. (2011). Shorter abstinence decreases sperm deoxyribonucleic acid fragmentation in ejaculate. Fertil. Steril..

[13] Agarwal A., Gupta S., Du Plessis S., Sharma R., Esteves S.C., Cirenza C., Eliwa J., Al-Najjar W., Kumaresan D., Haroun N. (2016). Abstinence Time and Its Impact on Basic and Advanced Semen Parameters. Urology.

[14] De Jonge C., LaFromboise M., Bosmans E., Ombelet W., Cox A., Nijs M. (2004). Influence of the abstinence period on human sperm quality. Fertil. Steril..

[15] Tvrdá E., Arroyo F., Ďuračka M., López-Fernández C., Gosálvez J. (2019). Dynamic assessment of human sperm DNA damage II: The effect of sperm concentration adjustment during processing. J. Assist. Reprod. Genet..

[16] Mayorga-Torres B.J., Camargo M., Agarwal A., du Plessis S.S., Cadavid Á.P., Cardona Maya W.D. (2015). Influence of ejaculation frequency on seminal parameters. Reprod. Biol. Endocrinol..

[17] Ayad B.M., Van der Horst G., du Plessis S.S. (2018). Short abstinence: A potential strategy for the improvement of sperm quality. Middle East Fertil. Soc. J..

[18] Sánchez-Martín P., Sánchez-Martín F., González-Martínez M., Gosálvez J. (2013). Increased pregnancy after reduced male abstinence. Syst. Biol. Reprod. Med..

[19] Wang H., Xu A., Gong L., Chen Z., Zhang B., Li X. (2022). The Microbiome, an Important Factor That Is Easily Overlooked in Male Infertility. Front. Microbiol..

[20] Allen-Vercoe E. (2013). Bringing the gut microbiota into focus through microbial culture: Recent progress and future perspective. Curr. Opin. Microbiol..

[21] Fraczek M., Hryhorowicz M., Gill K., Zarzycka M., Gaczarzewicz D., Jedrzejczak P., Bilinska B., Piasecka M., Kurpisz M. (2016). The effect of bacteriospermia and leukocytospermia on conventional and nonconventional semen parameters in healthy young normozoospermic males. J. Reprod. Immunol..

[22] Tvrdá E., Lovíšek D., Gálová E., Schwarzová M., Kováčiková E., Kunová S., Žiarovská J., Kačániová M. (2022). Possible Implications of Bacteriospermia on the Sperm Quality, Oxidative Characteristics, and Seminal Cytokine Network in Normozoospermic Men. Int. J. Mol. Sci..

[23] Perez-Carrasco V., Soriano-Lerma A., Soriano M., Gutiérrez-Fernández J., Garcia-Salcedo J.A. (2021). Urinary Microbiome: Yin and Yang of the Urinary Tract. Front. Cell. Infect. Microbiol..

[24] Gottschick C., Deng Z.L., Vital M., Masur C., Abels C., Pieper D.H., Wagner-Döbler I. (2017). The urinary microbiota of men and women and its changes in women during bacterial vaginosis and antibiotic treatment. Microbiome.

[25] Fouts D.E., Pieper R., Szpakowski S., Pohl H., Knoblach S., Suh M.J., Huang S.T., Ljungberg I., Sprague B.M., Lucas S.K. (2012). Integrated next-generation sequencing of 16S rDNA and metaproteomics differentiate the healthy urine microbiome from asymptomatic bacteriuria in neuropathic bladder associated with spinal cord injury. J. Transl. Med..

[26] Modena B.D., Milam R., Harrison F., Cheeseman J.A., Abecassis M.M., Friedewald J.J., Kirk A.D., Salomon D.R. (2017). Changes in Urinary Microbiome Populations Correlate in Kidney Transplants With Interstitial Fibrosis and Tubular Atrophy Documented in Early Surveillance Biopsies. Am. J. Transplant..

[27] Moustafa A., Li W., Singh H., Moncera K.J., Torralba M.G., Yu Y., Manuel O., Biggs W., Venter J.C., Nelson K.E. (2018). Microbial metagenome of urinary tract infection. Sci. Rep..

[28] Kogan M.I., Naboka Y.L., Ibishev K.S., Gudima I.A., Naber K.G. (2015). Human urine is not sterile—Shift of paradigm. Urol. Int..

[29] Gonçalves M.F.M., Fernandes Â.R., Rodrigues A.G., Lisboa C. (2022). Microbiome in Male Genital Mucosa (Prepuce, Glans, and Coronal Sulcus): A Systematic Review. Microorganisms.

[30] Lundy S.D., Sangwan N., Parekh N.V., Selvam M.K.P., Gupta S., McCaffrey P., Bessoff K., Vala A., Agarwal A., Sabanegh E.S. (2021). Functional and Taxonomic Dysbiosis of the Gut, Urine, and Semen Microbiomes in Male Infertility. Eur. Urol..

[31] Alqawasmeh O., Fok E., Yim H., Li T., Chung J., Chan D. (2022). The microbiome and male infertility: Looking into the past to move forward. Hum. Fertil..

[32] Stones D.H., Krachler A.M. (2016). Against the tide: The role of bacterial adhesion in host colonization. Biochem. Soc. Trans..

[33] Vander H., Gupta S., Kaur S., Kaur K., Prabha V. (2013). Characterization of sperm immobilization factor from Escherichia coli and its receptor to study the underlying mechanism of sperm immobilization. Am. J. Biomed Sci..

[34] Prabha V., Gupta T., Kaur S., Kaur N., Kala S., Singh A. (2009). Isolation of a spermatozoal immobilization factor from Staphylococcus aureus filtrates. Can. J. Microbiol..

[35] Berger G.K., Smith-Harrison L.I., Sandlow J.I. (2019). Sperm agglutination: Prevalence and contributory factors. Andrologia.

[36] Gioannini T.L., Weiss J.P. (2007). Regulation of interactions of Gramnegative bacterial endotoxins with mammalian cells. Immunol. Res..

[37] Fraczek M., Szumala-Kakol A., Jedrzejczak P., Kamieniczna M., Kurpisz M. (2007). Bacteria trigger oxygen radical release and sperm lipid peroxidation in in vitro model of semen inflammation. Fertil. Steril..

[38] Fraczek M., Szumala-Kakol A., Dworacki G., Sanocka D., Kurpisz M. (2013). In vitro reconstruction of inflammatory reaction in human semen: Effect on sperm DNA fragmentation. J. Reprod. Immunol..

[39] Loveland K.L., Klein B., Pueschl D., Indumathy S., Bergmann M., Loveland B.E., Hedger M.P., Schuppe H.C. (2017). Cytokines in Male Fertility and Reproductive Pathologies: Immunoregulation and Beyond. Front. Endocrinol..

[40] Boitrelle F., Shah R., Saleh R., Henkel R., Kandil H., Chung E., Vogiatzi P., Zini A., Arafa M., Agarwal A. (2021). The Sixth Edition of the WHO Manual for Human Semen Analysis: A Critical Review and SWOT Analysis. Life.

[41] Valsa J., Skandhan K.P., Gusani P.H., Sahab Khan P., Amith S. (2013). Quality of 4-hourly ejaculates--levels of calcium and magnesium. Andrologia.

[42] Küçük T., Sozen E., Buluc B. (2008). Intrauterine insemination with double ejaculate compared with single ejaculate in male factor infertility: A pilot study. J. Androl..

[43] Patil P.S., Humbarwadi R.S., Patil A.D., Gune A.R. (2013). Immature germ cells in semen—Correlation with total sperm count and sperm motility. J. Cytol..

[44] Comar V.A., Petersen C.G., Mauri A.L., Mattila M., Vagnini L.D., Renzi A., Petersen B., Nicoletti A., Dieamant F., Oliveira J.B.A. (2017). Influence of the abstinence period on human sperm quality: Analysis of 2,458 semen samples. JBRA Assist. Reprod..

[45] Dahan M.H., Mills G., Khoudja R., Gagnon A., Tan G., Tan S.L. (2021). Three hour abstinence as a treatment for high sperm DNA fragmentation: A prospective cohort study. J. Assist. Reprod. Genet..

[46] Agnihotri S.K., Agrawal A.K., Hakim B.A., Vishwakarma A.L., Narender T., Sachan R., Sachdev M. (2016). Mitochondrial membrane potential (MMP) regulates sperm motility. In Vitro Cell. Dev. Biol. Anim..

[47] Du Plessis S.S., Agarwal A., Halabi J., Tvrda E. (2015). Contemporary evidence on the physiological role of reactive oxygen species in human sperm function. J. Assist. Reprod. Genet..

[48] Tvrdá E., Benko F., Ďuračka M. (2022). Oxidative Stress as an Underlying Mechanism of Bacteria-Inflicted Damage to Male Gametes. Oxygen.

[49] Pahune P.P., Choudhari A.R., Muley P.A. (2013). The total antioxidant power of semen and its correlation with the fertility potential of human male subjects. J. Clin. Diagn. Res..

[50] Boe-Hansen G.B., Fedder J., Ersbøll A.K., Christensen P. (2006). The sperm chromatin structure assay as a diagnostic tool in the human fertility clinic. Hum. Reprod..

[51] Chen G.X., Li H.Y., Lin Y.H., Huang Z.Q., Huang P.Y., Da L.C., Shi H., Yang L., Feng Y.B., Zheng B.H. (2022). The effect of age and abstinence time on semen quality: A retrospective study. Asian J. Androl..

[52] Zandieh Z., Vatannejad A., Doosti M., Zabihzadeh S., Haddadi M., Bajelan L., Rashidi B., Amanpour S. (2018). Comparing reactive oxygen species and DNA fragmentation in semen samples of unexplained infertile and healthy fertile men. Ir. J. Med. Sci..

[53] Eini F., Kutenaei M.A., Zareei F., Dastjerdi Z.S., Shirzeyli M.H., Salehi E. (2021). Effect of bacterial infection on sperm quality and DNA fragmentation in subfertile men with leukocytospermia. BMC Mol. Cell. Biol..

[54] Enwuru C.A., Iwalokun B., Enwuru V.N., Ezechi O., Oluwadun A. (2016). The effect of presence of facultative bacteria species on semen and sperm quality of men seeking fertility care. Afr. J. Urol..

[55] Vilvanathan S., Kandasamy B., Jayachandran A.L., Sathiyanarayanan S., Tanjore Singaravelu V., Krishnamurthy V., Elangovan V. (2016). Bacteriospermia and its impact on basic semen parameters among infertile men. Interdiscip. Perspect. Infect. Dis..

[56] Voroshilina E.S., Zornikov D.L., Ivanov A.V., Pochernikov D.G., Panacheva E.A. (2021). Microbiota of semen samples with normozoospermia: Analysis of real-time PCR data. Bull. Russ. State Med. Univ..

[57] Hou D., Zhou X., Zhong X., Settles M.L., Herring J., Wang L., Abdo Z., Forney L.J., Xu C. (2013). Microbiota of the seminal fluid from healthy and infertile men. Fertil. Steril..

[58] Jedrzejczak P., Fraczek M., Szumała-Kakol A., Taszarek-Hauke G., Pawelczyk L., Kurpisz M. (2005). Consequences of semen inflammation and lipid peroxidation on fertilization capacity of spermatozoa in vitro conditions. Int. J. Androl..

[59] Zhang F., Dai J., Chen T. (2021). Role of Lactobacillus in female infertility via modulating sperm agglutination and immobilization. Front. Cell. Infect. Microbiol..

[60] Wolff H., Panhans A., Stolz W., Meurer M. (1993). Adherence of Escherichia coli to sperm: A mannose mediated phenomenon leading to agglutination of sperm and E. coli. Fertil. Steril..

[61] Rennemeier C., Frambach T., Hennicke F., Dietl J., Staib P. (2009). Microbial quorum-sensing molecules induce acrosome loss and cell death in human spermatozoa. Infect. Immun..

[62] Shang Y., Liu C., Cui D., Han G., Yi S. (2014). The effect of chronic bacterial prostatitis on semen quality in adult men: A meta-analysis of case-control studies. Sci. Rep..

[63] Wang S., Zhang K., Yao Y., Li J., Deng S. (2021). Bacterial infections affect male fertility: A focus on the oxidative stress-autophagy axis. Front. Cell. Dev. Biol..

[64] Benoff S., Cooper G.W., Centola G.M., Jacob A., Hershlag A., Hurley I.R. (2000). Metal ions and human sperm mannose receptors. Andrologia.

[65] Agarwal J., Srivastava S., Singh M. (2012). Pathogenomics of uropathogenic Escherichia coli. Indian J. Med. Microbiol..

[66] Pant N.C., Singh R., Gupta V., Chauhan A., Mavuduru R., Prabha V., Sharma P. (2019). Contraceptive efficacy of sperm agglutinating factor from Staphylococcus warneri, isolated from the cervix of a woman with inexplicable infertility. Reprod. Biol. Endocrinol..

[67] Henderson B., Martin A. (2011). Bacterial virulence in the moonlight: Multitasking bacterial moonlighting proteins are virulence determinants in infectious disease. Infect. Immun..

[68] Moretti E., Capitani S., Figura N., Pammolli A., Federico M.G., Giannerini V., Collodel G. (2009). The presence of bacterial species in semen and sperm quality. J. Assisted Reprod. Genet..

[69] Domes T., Lo K.C., Grober E.D., Mullen J.B., Mazzulli T., Jarvi K. (2012). The incidence and effect of bacteriospermia and elevated seminal leukocytes on semen parameters. Fertil. Steril..

[70] Berjis K., Ghiasi M., Sangy S. (2018). Study of seminal infection among an infertile male population in Qom, Iran, and its effect on sperm quality. Iran. J. Microbiol..

[71] Fraczek M., Piasecka M., Gaczarzewicz D., Szumala-Kakol A., Kazienko A., Lenart S., Laszczynska M., Kurpisz M. (2012). Membrane stability and mitochondrial activity of human-ejaculated spermatozoa during in vitro experimental infection with Escherichia coli, Staphylococcus haemolyticus and Bacteroides ureolyticus. Andrologia.

[72] Schulz M., Sánchez R., Soto L., Risopatrón J., Villegas J. (2010). Effect of Escherichia coli and its soluble factors on mitochondrial membrane potential, phosphatidylserine translocation, viability, and motility of human spermatozoa. Fertil. Steril..

[73] Sanocka D., Fraczek M., Jedrzejczak P., Szumała-Kakol A., Kurpisz M. (2004). Male genital tract infection: An influence of leukocytes and bacteria on semen. J. Reprod. Immunol..

[74] Rusz A., Pilatz A., Wagenlehner F., Linn T., Diemer T., Schuppe H.C., Lohmeyer J., Hossain H., Weidner W. (2012). Influence of urogenital infections and inflammation on semen quality and male fertility. World J. Urol..

[75] Fraczek M., Kurpisz M. (2015). Mechanisms of the harmful effects of bacterial semen infection on ejaculated human spermatozoa: Potential inflammatory markers in semen. Folia Histochem. Cytobiol..

[76] Martínez P., Proverbio F., Camejo M.I. (2007). Sperm lipid peroxidation and pro-inflammatory cytokines. Asian J. Androl..

[77] Perdichizzi A., Nicoletti F., La Vignera S., Barone N., D’Agata R., Vicari E., Calogero A.E. (2007). Effects of tumour necrosis factor-alpha on human sperm motility and apoptosis. J. Clin. Immunol..

[78] Paira D.A., Silvera-Ruiz S., Tissera A., Molina R.I., Olmedo J.J., Rivero V.E., Motrich R.D. (2022). Interferon γ, IL-17, and IL-1β impair sperm motility and viability and induce sperm apoptosis. Cytokine.

[79] Dziadecki W., Celińska A., Fracki S., Bablok L., Barcz E. (2010). Interleukin 1b and interleukin 18 and their connection with leukocytospermia in human semen. Centr. Eur. J. Immunol..

[80] Koçak I., Yenisey C., Dündar M., Okyay P., Serter M. (2002). Relationship between seminal plasma interleukin-6 and tumor necrosis factor alpha levels with semen parameters in fertile and infertile men. Urol. Res..

[81] Moretti E., Collodel G., Mazzi L., Campagna M., Iacoponi F., Figura N. (2014). Resistin, interleukin-6, tumor necrosis factor-alpha, and human semen parameters in the presence of leukocytospermia, smoking habit, and varicocele. Fertil. Steril..

[82] Zeyad A., Hamad M.F., Hammadeh M.E. (2018). The effects of bacterial infection on human sperm nuclear protamine P1/P2 ratio and DNA integrity. Andrologia.

[83] Skandhan K.P. (2004). Hypothesis: Epididymis inhibits sperm motility inside male reproductive tract. Med. Hypotheses.

[84] Tvrdá E., Arroyo F., Gosálvez J. (2018). Dynamic assessment of human sperm DNA damage I: The effect of seminal plasma-sperm co-incubation after ejaculation. Int. Urol. Nephrol..

[85] Lu Z., Sethu R., Imlay J.A. (2018). Endogenous superoxide is a key effector of the oxygen sensitivity of a model obligate anaerobe. Proc. Natl. Acad. Sci. USA.

[86] Chakraborty S.P., Pramanik P., Roy S. (2012). Staphylococcus aureus Infection induced oxidative imbalance in neutrophils: Possible protective role of nanoconjugated vancomycin. ISRN Pharmacol..

[87] Duracka M., Lukac N., Kacaniova M., Kantor A., Hleba L., Ondruska L., Tvrda E. (2019). Antibiotics Versus Natural Biomolecules: The Case of In Vitro Induced Bacteriospermia by Enterococcus Faecalis in Rabbit Semen. Molecules.

[88] Barroso G., Morshedi M., Oehninger S. (2000). Analysis of DNA fragmentation, plasma membrane translocation of phosphatidylserine and oxidative stress in human spermatozoa. Hum. Reprod..

[89] Collodel G., Baccetti B., Capitani S., Moretti E. (2007). Necrosis in human spermatozoa. I. Ultrastructural features and FISH study in semen from patients with urogenital infections. J. Submicrosc. Cytol. Pathol..

[90] Sharma R.K., Seifarth K., Agarwal A. (1997). Comparison of single- and two-layer Percoll separation for selection of motile spermatozoa. Int. J. Fertil. Womens Med..

[91] Cui Z., Sharma R., Agarwal A. (2016). Proteomic analysis of mature and immature ejaculated spermatozoa from fertile men. Asian J. Androl..

[92] Tvrda E., Fik M., Kovacik A., Duracka M., Kacaniova M. (2022). Biochemical and bacteriological characterization of Dachshund semen: A correlation study. Reprod. Domest. Anim..

[93] Schuffner A., Morshedi M., Oehninger S. (2001). Cryopreservation of fractionated, highly motile human spermatozoa: Effect on membrane phosphatidylserine externalization and lipid peroxidation. Hum. Reprod..

[94] Tvrdá E., López-Fernández C., Sánchez-Martín P., Gosálvez J. (2018). Sperm DNA fragmentation in donors and normozoospermic patients attending for a first spermiogram: Static and dynamic assessment. Andrologia.

[95] Muller C.H., Lee T.K., Montaño M.A. (2013). Improved chemiluminescence assay for measuring antioxidant capacity of seminal plasma. Methods Mol. Biol..

[96] Weber D., Davies M.J., Grune T. (2015). Determination of Protein Carbonyls in Plasma, Cell Extracts, Tissue Homogenates, Isolated Proteins: Focus on Sample Preparation and Derivatization Conditions. Redox Biol..

[97] Tvrdá E., Kačániová M., Baláži A., Vašíček J., Vozaf J., Jurčík R., Ďuračka M., Žiarovská J., Kováč J., Chrenek P. (2022). The Impact of Bacteriocenoses on Sperm Vitality, Immunological and Oxidative Characteristics of Ram Ejaculates: Does the Breed Play a Role?. Animals.

[98] Tvrdá E., Lovíšek D., Kyzek S., Kováčik D., Gálová E. (2021). The Effect of Non-Thermal Plasma on the Structural and Functional Characteristics of Human Spermatozoa. Int. J. Mol. Sci..

[99] Benko F., Fialková V., Žiarovská J., Ďuračka M., Lukáč N., Tvrdá E. (2022). In Vitro versus Cryo-Induced Capacitation of Bovine Spermatozoa, Part 2: Changes in the Expression Patterns of Selected Transmembrane Channels and Protein Kinase A. Int. J. Mol. Sci..

