# Supplementary material for: Ejaculatory Abstinence Affects the Sperm Quality in Normozoospermic Men—How Does the Seminal Bacteriome Respond?
Source: Int J Mol Sci. 2023 Feb 9;24(4):3503. doi: 10.3390/ijms24043503 (PMC9963725; doi:10.3390/ijms24043503)
Supplement: Supplementary file 1 [file ijms-24-03503-s001.zip › ijms-2156680-supplementary.pdf]

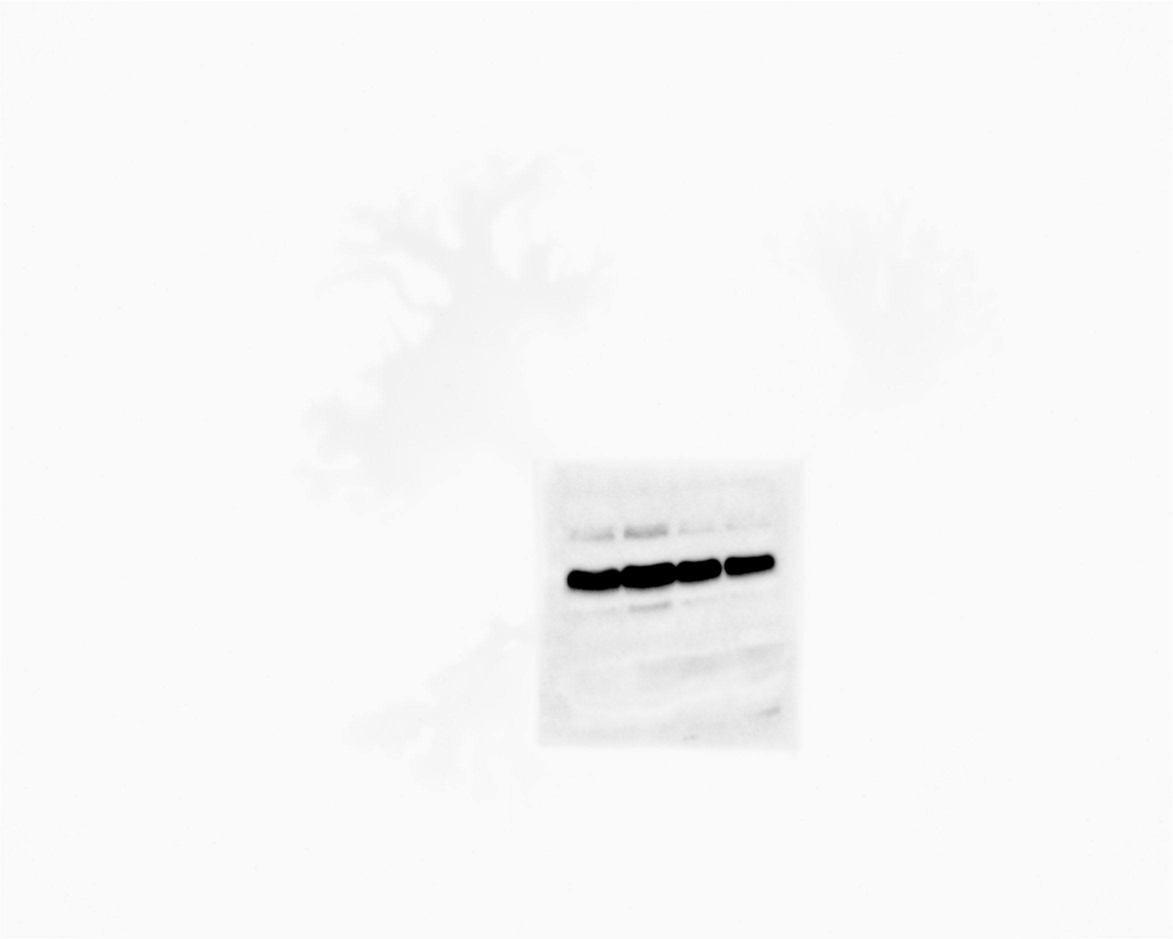

Figure S1. Original blot of BAX protein.

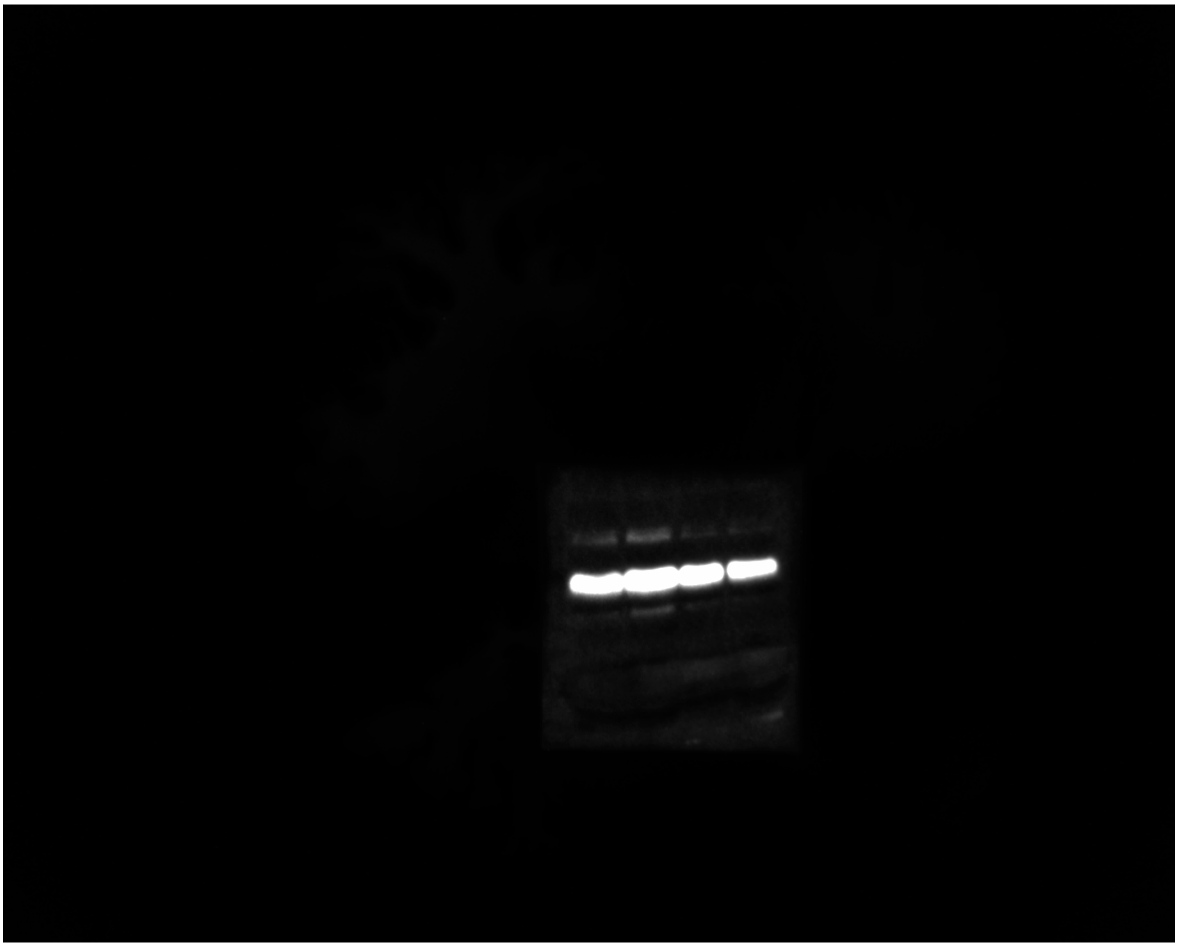

Figure S2. Inverted blot of BAX protein.

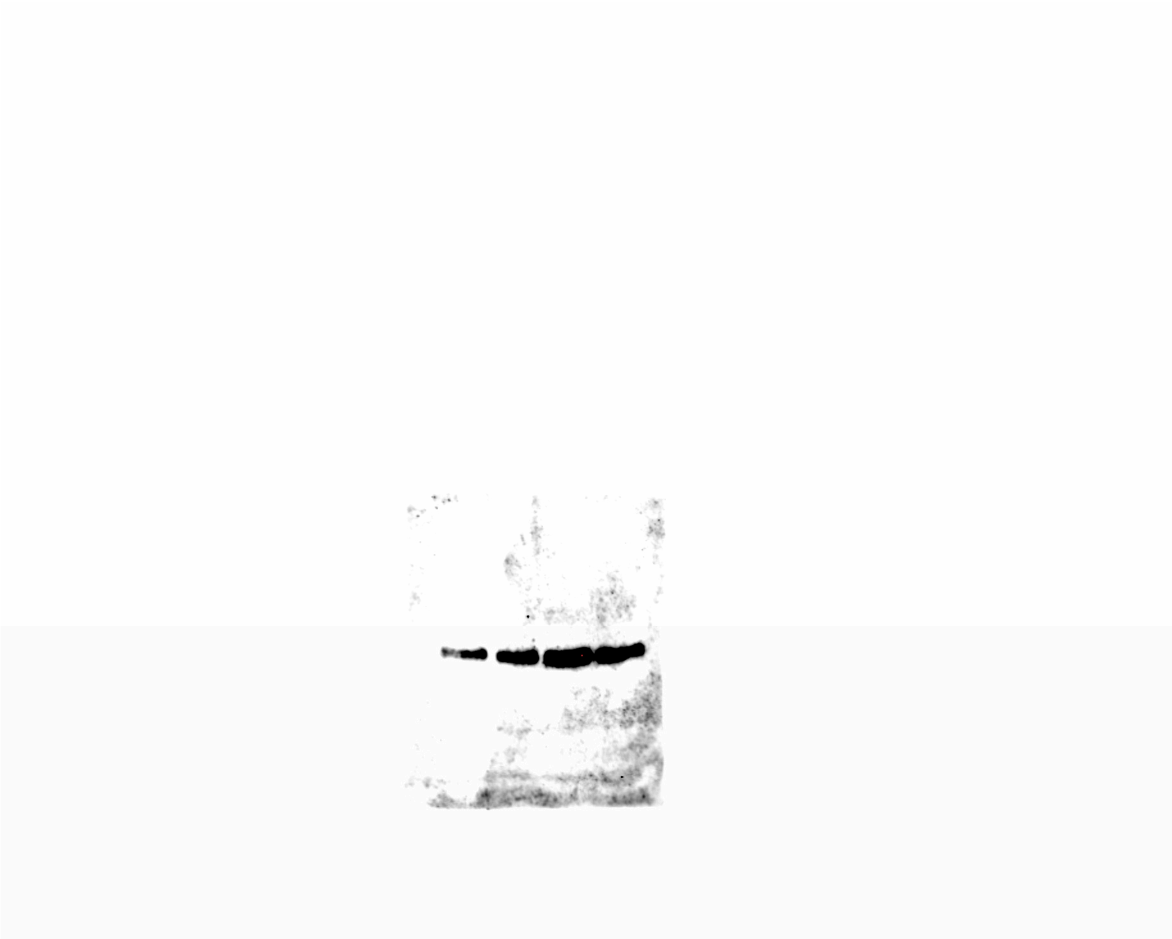

Figure S3. Original blot of Bcl-2 protein.

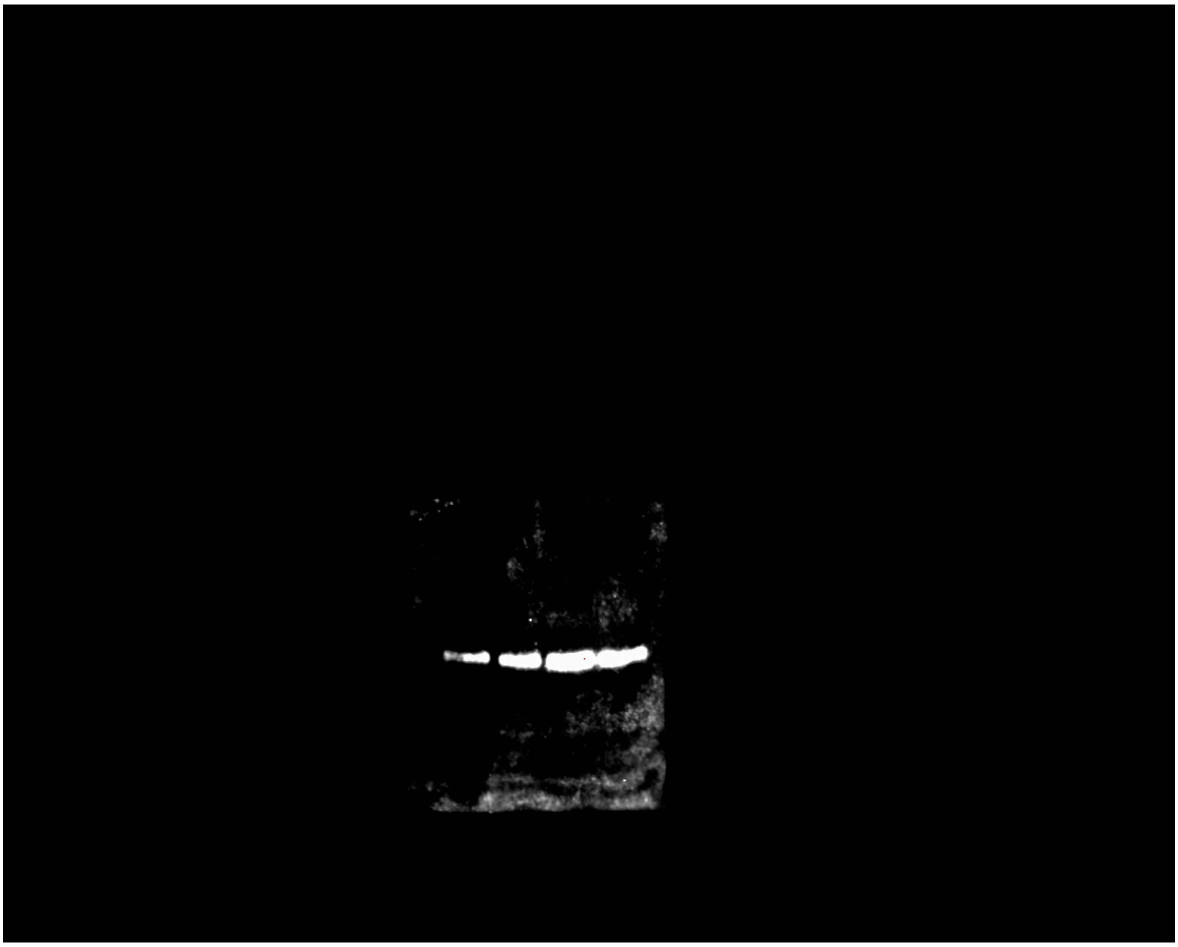

Figure S4. Inverted blot of Bcl-2 protein.

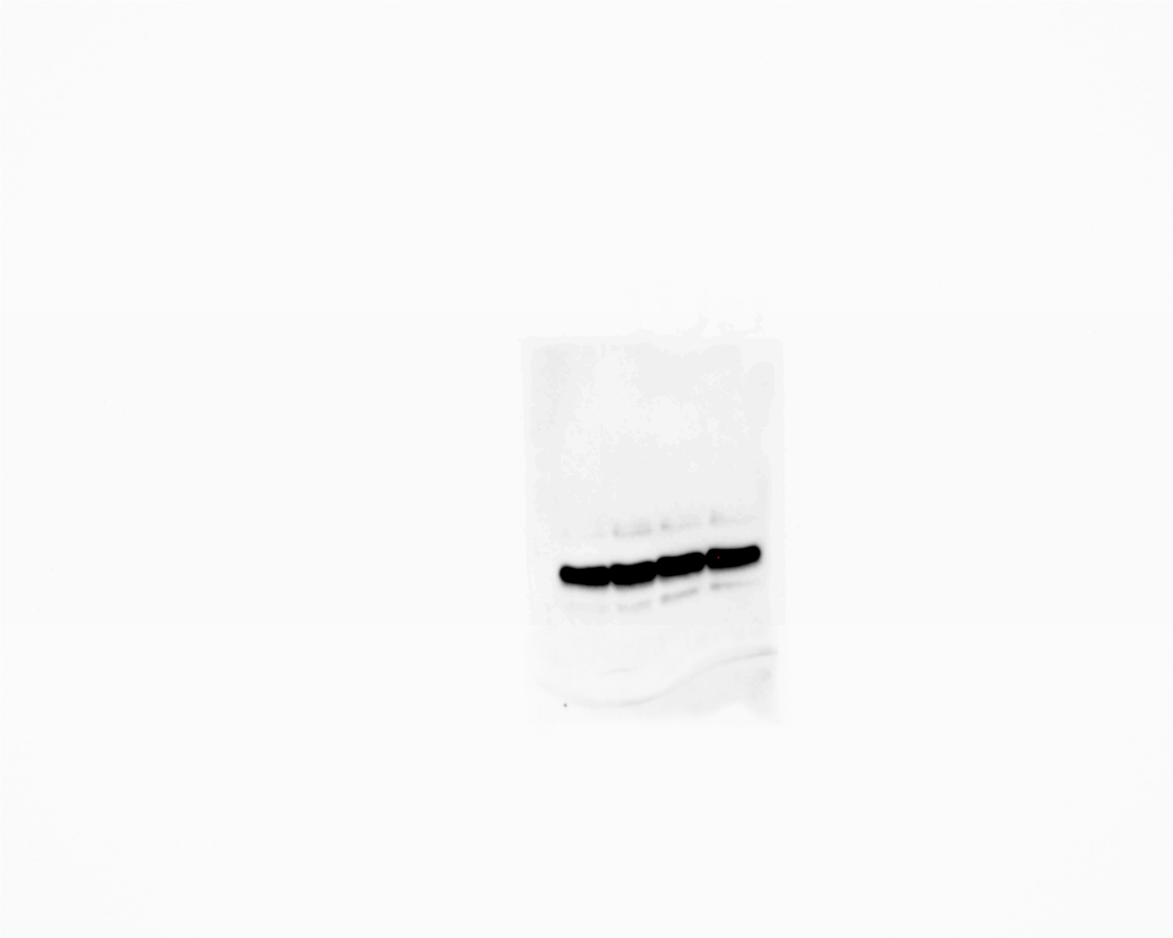

Figure S5. Original blot of beta actin.

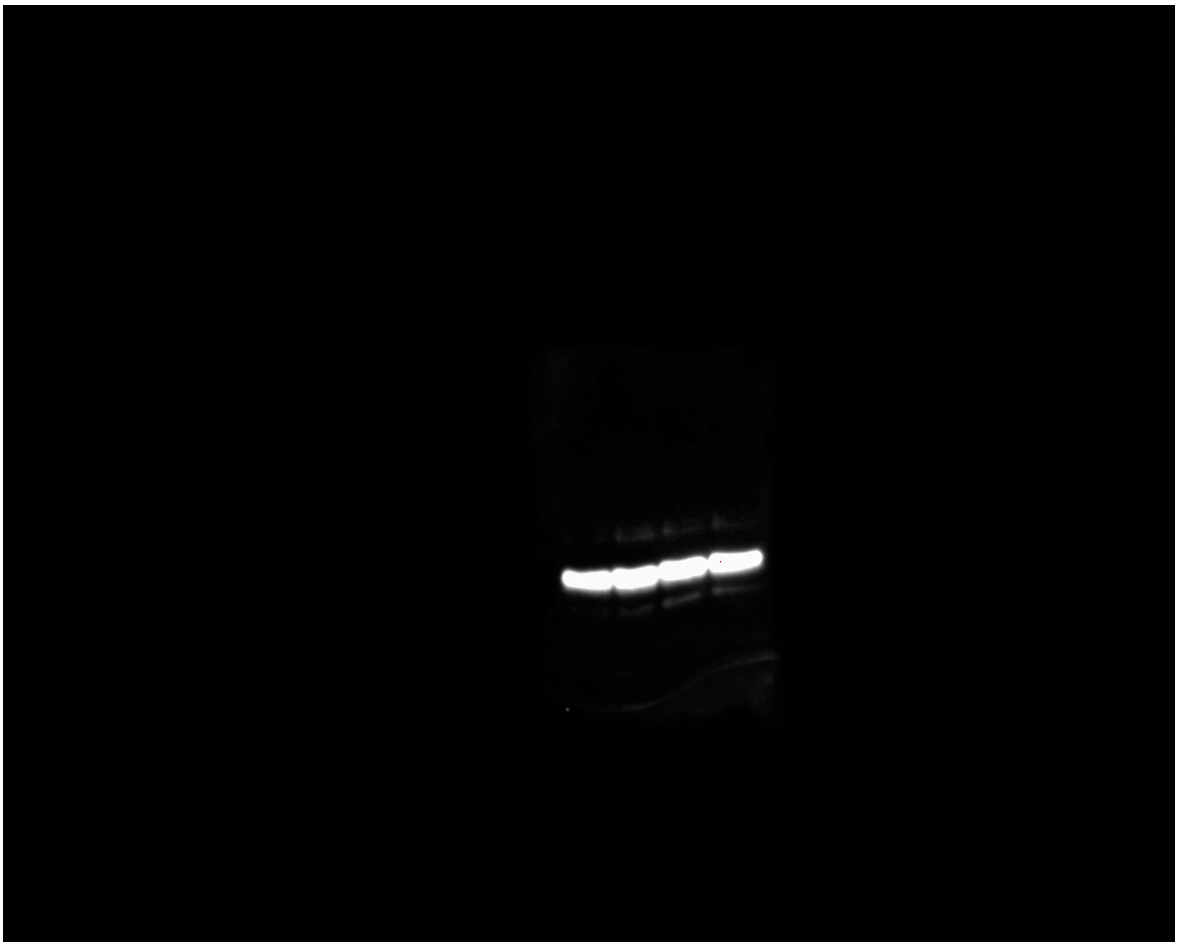

Figure S6. Inverted blot of beta actin.
